# Predicting cellular responses to complex perturbations in high‐throughput screens

**DOI:** 10.15252/msb.202211517

**Published:** 2023-05-08

**Authors:** Mohammad Lotfollahi, Anna Klimovskaia Susmelj, Carlo De Donno, Leon Hetzel, Yuge Ji, Ignacio L Ibarra, Sanjay R Srivatsan, Mohsen Naghipourfar, Riza M Daza, Beth Martin, Jay Shendure, Jose L McFaline‐Figueroa, Pierre Boyeau, F Alexander Wolf, Nafissa Yakubova, Stephan Günnemann, Cole Trapnell, David Lopez‐Paz, Fabian J Theis

**Affiliations:** ^1^ Helmholtz Center Munich – German Research Center for Environmental Health, Institute of Computational Biology Munich Germany; ^2^ Wellcome Trust Sanger Institute, Wellcome Genome Campus, Hinxton Cambridgeshire UK; ^3^ Meta AI Paris France; ^4^ Swiss Data Science Center Zurich Switzerland; ^5^ School of Life Sciences Weihenstephan Technical University of Munich Munich Germany; ^6^ Department of Mathematics Technical University of Munich Munich Germany; ^7^ Department of Genome Sciences University of Washington Seattle WA USA; ^8^ Department of Bioengineering University of California Berkeley CA USA; ^9^ Howard Hughes Medical Institute Seattle WA USA; ^10^ Brotman Baty Institute for Precision Medicine Seattle WA USA; ^11^ Allen Discovery Center for Cell Lineage Tracing Seattle WA USA; ^12^ Department of Biomedical Engineering Columbia University New York NY USA; ^13^ Department of Electrical Engineering and Computer Sciences University of California Berkeley CA USA; ^14^ Department of Computer Science Technical University of Munich Munich Germany; ^15^ Present address: Lamin Labs Munich Germany

**Keywords:** generative modeling, high‐throughput screening, machine learning, perturbation prediction, single‐cell transcriptomics, Computational Biology, Methods & Resources

## Abstract

Recent advances in multiplexed single‐cell transcriptomics experiments facilitate the high‐throughput study of drug and genetic perturbations. However, an exhaustive exploration of the combinatorial perturbation space is experimentally unfeasible. Therefore, computational methods are needed to predict, interpret, and prioritize perturbations. Here, we present the compositional perturbation autoencoder (CPA), which combines the interpretability of linear models with the flexibility of deep‐learning approaches for single‐cell response modeling. CPA learns to *in silico* predict transcriptional perturbation response at the single‐cell level for unseen dosages, cell types, time points, and species. Using newly generated single‐cell drug combination data, we validate that CPA can predict unseen drug combinations while outperforming baseline models. Additionally, the architecture's modularity enables incorporating the chemical representation of the drugs, allowing the prediction of cellular response to completely unseen drugs. Furthermore, CPA is also applicable to genetic combinatorial screens. We demonstrate this by imputing *in silico* 5,329 missing combinations (97.6% of all possibilities) in a single‐cell Perturb‐seq experiment with diverse genetic interactions. We envision CPA will facilitate efficient experimental design and hypothesis generation by enabling *in silico* response prediction at the single‐cell level and thus accelerate therapeutic applications using single‐cell technologies.

## Graphical abstract

The compositional perturbation autoencoder (CPA) is a deep learning model for predicting the transcriptomic responses of single cells to single or combinatorial treatments from drugs and genetic manipulations.

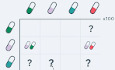

## Introduction

Single‐cell RNA‐sequencing (scRNA‐seq) profiles gene expression in millions of cells across tissues (The Tabula Muris Consortium, 2019; Domcke *et al*, 2020) and species (Han *et al*, 2020). Recently, novel technologies have been developed extending these measurements to high‐throughput screens (HTSs), which measure response to thousands of independent perturbations (Norman *et al*, 2019; Srivatsan *et al*, 2020). These advances show promise for facilitating and accelerating drug development (Yofe *et al*, 2020). HTSs applied at the single‐cell level provide both comprehensive molecular phenotyping and capture heterogeneous responses, which otherwise could not be identified using traditional HTSs (Srivatsan *et al*, 2020).

While the development of high‐throughput approaches such as “cellular hashing” (McGinnis *et al*, 2019; Gehring *et al*, 2020; Srivatsan *et al*, 2020; preprint: Martin *et al*, 2021) facilitates scRNA‐seq in multi‐sample experiments at low cost, these strategies require expensive library preparation (Srivatsan *et al*, 2020) and do not easily scale to large numbers of perturbations. These shortcomings become more apparent when exploring the effects of combination therapies (Al‐Lazikani *et al*, 2012; Kim *et al*, 2016; Sachs *et al*, 2020) or genetic perturbations (Dixit *et al*, 2016; Datlinger *et al*, 2017; Norman *et al*, 2019), where the experimental screening of all possible combinations becomes infeasible. While projects such as the Human Cell Atlas (Rozenblatt‐Rosen *et al*, 2017) aim to comprehensively map cellular states across tissues in a reproducible fashion, the construction of a similar atlas for the effects of perturbations on gene expression is impossible due to the vast number of possibilities. Since brute‐force exploration of the combinatorial search space is infeasible, it is necessary to develop computational tools to guide the exploration of the combinatorial perturbation space to nominate promising candidate combination therapies in HTSs. A successful computational method for the navigation of the combinatorial space must be able to predict the behavior of cells when subject to novel combinations of perturbations only measured separately in the original experiment. These data are referred to as Out‐Of‐Distribution (OOD) data. OOD prediction would enable the study of perturbations in the presence of different treatment doses (Hagai *et al*, 2018; Srivatsan *et al*, 2020), combination therapies (Gehring *et al*, 2020), multiple genetic knockouts (Norman *et al*, 2019), and changes across time (Hagai *et al*, 2018).

Recently, several computational approaches have been developed for predicting cellular responses to perturbations (Fröhlich *et al*, 2018; Lotfollahi *et al*, 2019, 2020; Rampášek *et al*, 2019; Yuan *et al*, 2021). The first approach leverages mechanistic modeling (Fröhlich *et al*, 2018; Yuan *et al*, 2021) to predict cell viability (Fröhlich *et al*, 2018) or the abundance of a few selected proteins (Yuan *et al*, 2021). Although they are powerful at interpreting interactions, mechanistic models usually require longitudinal data (which is often unavailable in practice) and most do not scale to genome wide measurements to predict high‐dimensional scRNA‐seq data. Linear models (Dixit *et al*, 2016; Kamimoto *et al*, 2023) do not suffer from these scalability issues, but have limited predictive power and are unable to capture nonlinear cell‐type‐specific responses. In contrast, deep learning (DL) models do not face these limitations. Recently, DL methods have been used to model gene expression latent spaces from scRNA‐seq data (Lopez *et al*, 2018, 2020; Eraslan *et al*, 2019; Lotfollahi *et al*, 2022), to describe and predict single‐cell responses (Lotfollahi *et al*, 2019, 2020; Rampášek *et al*, 2019; Russkikh *et al*, 2020). However, current DL‐based approaches also have limitations: they model only a handful of perturbations; cannot handle combinatorial treatments; and cannot incorporate continuous covariates such as dose and time, or discrete covariates such as cell types, species, and patients. Therefore, while current DL methods have modeled individual perturbations, none have been proposed for HTS.

Here, we propose the compositional perturbation autoencoder (CPA), a method to predict scRNA‐seq perturbation responses across combinations of conditions such as dosage, time, drug, and genetic knock‐out. The CPA borrows ideas from interpretable linear models and applies them in a flexible DL model to learn factorized latent representations of both perturbations and covariates. Given a scRNA‐seq dataset, the perturbations applied, and covariates describing the experimental setting, CPA decomposes the data into a collection of embeddings (representations) associated with the cell type, perturbation, and other external covariates. By virtue of an adversarial loss, these embeddings are independent of each other, so they can be recombined at prediction time to predict the effect of novel perturbation‐covariate combinations. Therefore, by exploring novel combinations, CPA can guide experimental design by directing hypotheses toward expression patterns of interest to experimentalists. We demonstrate the usefulness of CPA on six public datasets and a novel non‐small cell lung cancer (A549) dataset comprised of 32 single and combinatorial drug perturbations across multiple tasks, including the prediction and analysis of responses to compounds, doses, time‐series information, and genetic perturbations.

## Results

### Multiple perturbations as compositional processes in gene expression latent space

Prior work has modeled the effects of perturbations on gene expression separate processes. While differential expression compares each condition separately with a control, modeling a joint latent space with a conditional variational autoencoder (Sohn *et al*, 2015; Lotfollahi *et al*, 2020; Russkikh *et al*, 2020) is highly uninterpretable and not amenable to the prediction of the effects of combinations of conditions. Our goal here is to factorize the latent space of neural networks to turn them into interpretable, compositional models. If the latent space were linear, we could describe the observed gene expression as a factor model where each component is a single perturbation.

However, gene expression latent spaces, particularly in complex tissues, are nonlinear and best described by a graph or nonlinear embedding approximations (van der Maaten & Hinton, 2008; preprint: McInnes *et al*, 2018). In scRNA‐seq datasets, gene expression profiles of cell populations are often observed under multiple perturbations such as drugs, genetic knockouts, or disease states, in labeled covariates such as cell line, patient, or species. Each cell is labeled with its experimental condition and perturbation, where experimental covariates are captured in categorical labels and perturbations are captured using a continuous value (e.g., a drug applied with different doses). This assumes a sufficient number of cells per condition to permit the estimation of the latent space in control and perturbation states using a large neural network.

Instead of assuming a factor model in gene expression space, we instead model the nonlinear superposition of perturbation effects in the nonlinear latent space, in which we constrain the superposition to be additive (see Materials and Methods). We decouple the effects of perturbations and covariates, and allow for continuous effects such as drug dose by encoding this information in a nonlinearly transformed scalar weight: a learned drug‐response curve. The linear latent space factor model enables interpretation of this space by disentangling latent space variance driven by covariates from those stemming from each perturbation. At evaluation time, we are able to not only interpolate and interpret the observed perturbation combinations, but also to predict other combinations, potentially in different covariate settings.

### Compositional perturbation autoencoder (CPA)

**Figure 1:**
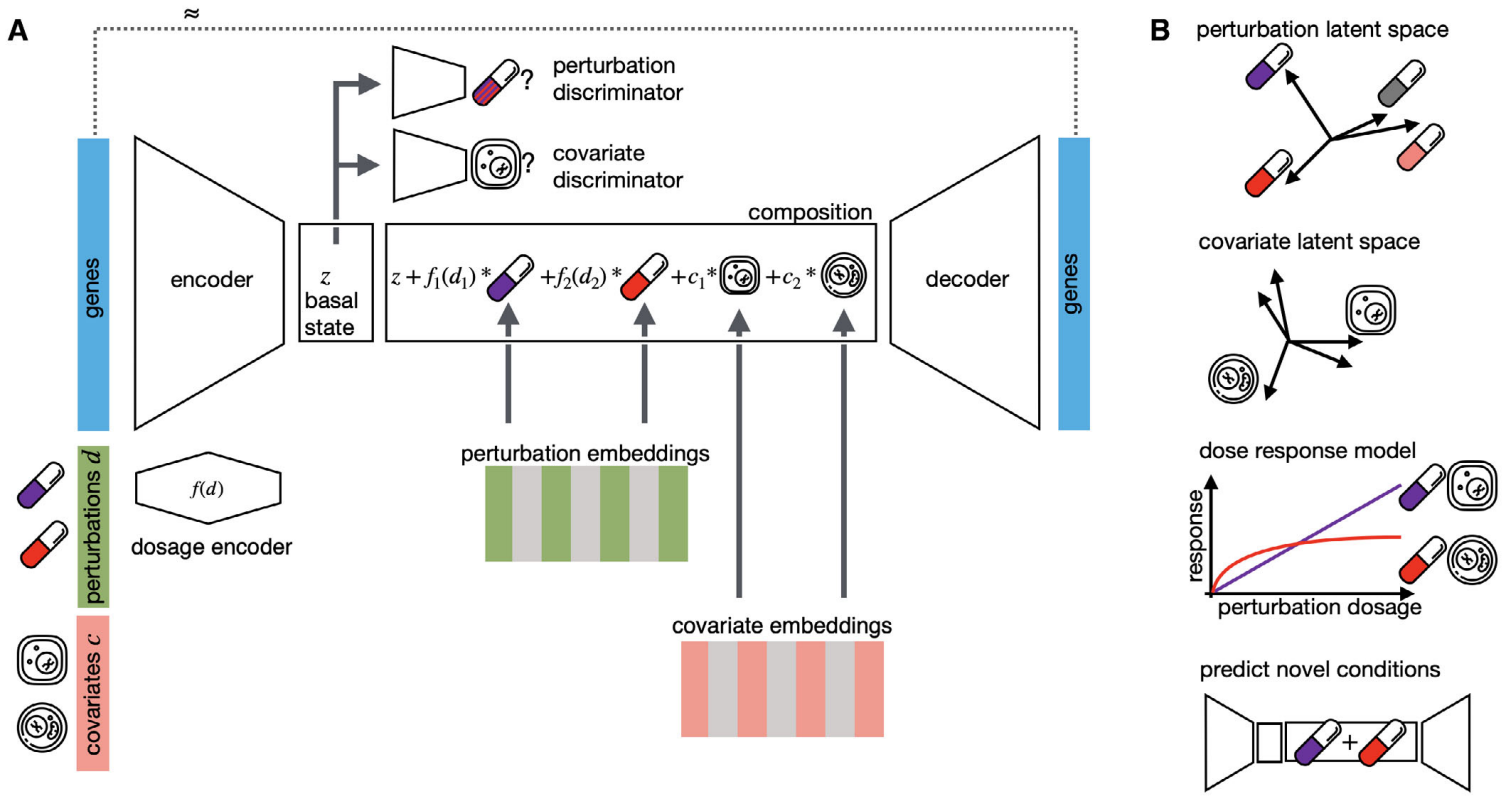
Interpretable single‐cell perturbation modeling using a compositional perturbation autoencoder (CPA) Given a matrix of gene expression per cell together with annotated potentially quantitative perturbations d and other covariates such as cell line, patient, or species, CPA learns the combined perturbation response for a single‐cell. It encodes gene expression using a neural network into a lower dimensional latent space that is eventually decoded back to an approximate gene expression matrix, as close as possible to the original one. To make the latent space interpretable in terms of perturbation and covariates, the encoded gene expression vector is first mapped to a “basal state” by feeding the signal to discriminators to remove any signal from perturbations and covariates. The basal state is then composed with perturbations and covariates, with potentially reweighted dosages, to reconstruct the gene expression. All encoder, decoder, and discriminator weights as well as the perturbation and covariate dictionaries are learned during training.Features of CPA are interpreted via plotting of the two learned dictionaries, interpolating covariate‐specific dose–response curves and predicting novel unseen drug combinations.

We introduce the CPA (see Materials and Methods), a method combining ideas from natural language processing (preprint: Mikolov *et al*, 2013) and computer vision (preprint: Radford *et al*, 2015; Lample *et al*, 2017) to predict the effects of combinations of perturbations on single‐cell gene expression. Given a single‐cell dataset of multiple perturbations and covariates, the CPA first uses an encoder neural network to decompose the cells' gene expression into a series of learnable, additive embeddings, which correspond to its basal state, the observed perturbation, and the observed covariates. Crucially, the latent representation that the CPA encoder learns about a cell's basal state is disentangled from (does not contain information about) the embeddings corresponding to the perturbation and the covariates. This disentangling is achieved by training a discriminator classifier (Lample *et al*, 2017; Zhao *et al*, 2018) in a competition against the encoder network of the CPA. The goal of the encoder network in the CPA is to learn an embedding representing the cell's basal state, from which the discriminator network cannot predict the perturbation or covariate values. To account for continuous time or dose effects, the learned embeddings about each perturbation are scaled nonlinearly via a neural network which receives the continuous covariate values for each cell, such as the time or the dose. After the linear integration of the learned embeddings about the cell's basal state, perturbations, and covariate values into an unified embedding, the CPA uses a non‐linear neural network decoder to recover the cell's gene expression vector (Fig 1A and B, see Appendix Fig S1 and Materials and Methods for more details). The non‐linearity of the decoded enables capturing complex cell‐type‐specific and non‐additive effects of combinatorial treatments. Consider a simple example when a cell is perturbed with two gene knock‐out perturbations. CPA learns to reconstruct combinatorial treatment's overall gene expression effect via linearly combining singleton treatment embedding for each perturbation and the basal state fed to the decoder. Such a constraint allows the model to learn a pattern of how a single treatment behaves when combined with other treatments and thus enabling the prediction of combinations not seen during the training. The prediction performance for combinations of a given single perturbation could improve when the model observes diverse training data from that perturbation combined with others. Conversely, when the model has never seen training data containing combinations for a specific single perturbation, it could generate spurious predictions for combinations including that treatment.

Similar to many neural network models, the CPA is trained using backpropagation (Goodfellow *et al*, 2016) on the reconstruction and discriminator errors (see Materials and Methods), to tune the parameters of the encoder network, the decoder network, the embeddings corresponding to each perturbation and covariate value, and the dose/time nonlinear scalers. The learned embeddings allow the measurement of similarities between different perturbations and covariates, in terms of their effects on gene expression. The main feature of the CPA is its flexibility of use at evaluation time. After obtaining the disentangled embeddings corresponding to some observed gene expression, perturbation, and covariate values, we can intervene and swap the perturbation embedding with any other perturbation embedding of our choice. This manipulation is effectively a way of estimating the answer to the counterfactual question: what would the gene expression of this cell have looked like, had it been treated differently? This approach is of particular interest in the prediction of unseen perturbation combinations and their effects on gene expression. The CPA can also visualize the transcriptional similarity and uncertainty associated with perturbations and covariates, as later demonstrated.

### CPA allows predictive and exploratory analyses of single‐cell perturbation experiments

We first demonstrated the performance and functionality of the CPA on three small single‐cell datasets: a dataset of PBMCs stimulated with IFN‐*β* (Kang *et al*, 2018), a dataset of human lung cancer cells perturbed by four drugs (Srivatsan *et al*, 2020), and a longitudinal cross‐species dataset of lipopolysaccharide (LPS) treated phagocytes (Hagai *et al*, 2018; see Materials and Methods). The datasets represent different potential applications of the model: (i) binary perturbation in distinct cell types, (ii) diverse doses; and (iii) several species and variation with respect to time instead of dose. We split each dataset into three groups: train (used for model training), test (used for tuning the model parameters), and OOD (never seen during training or parameter setting, and intended to measure the generalization properties of the model).

Here, we considered PBMCs from lupus patient samples that were treated with IFN‐*β* (Appendix Fig S2A). The stimulation, in this case, is a binary one, without any continuous covariate (e.g., dosage or time) associated with it. In order to assess that CPA is capable of decoupling covariate and perturbation information when this is provided we trained two models: (i) one to which only perturbation labels were provided during training, (ii) and one to which both perturbation and cell type labels were provided. We then inspected the basal latent representation obtained with these models, this is the latent information remaining after covariate and/or perturbation information is transferred to the respective embeddings by means of adversarial training. As expected, the latent representation obtained with model (i) shows a good mixing of the perturbations while cell type information is retained (Appendix Fig S2B), on the other hand, the latent values obtained from model (ii) show good mixing of both cell types and perturbations, since in this case labels for both were provided to the model which successfully embedded this information in the correspondent latent factors (Appendix Fig S2C). In order to further demonstrate the differences between the two models we decoded the basal latent representation of B cells without factoring the cell type and perturbations embeddings and looked at the gene expression of *CD74* and *CD37*, two marker genes of B cells in this dataset (Appendix Fig S2D). The cells thus obtained from model (i) show high values of expression for these genes, this happens because cell type information is not decoupled and is retained in the basal latent space, this is not the case for model (ii) where cell type information has been successfully removed from the basal latent space (Appendix Fig S2E).

Furthermore, we leveraged this dataset to assess that the model is capable of learning cell‐type‐specific responses to perturbations. We trained different CPA models holding out perturbed cells belonging to one cell type at a time and then predicted the gene expression of the perturbed cells in these OOD conditions. CPA successfully models the response of various genes to stimulation in different cell types, even in the case of cell‐type‐specific responses (Appendix Fig S3). Sciplex2 from Srivatsan *et al* (2020) contains measurements of a human lung adenocarcinoma cell line (A549) treated with four drug perturbations at increasing dosages (Appendix Fig S4A). In this scenario, the model learns to generalize to unseen dosages of the drugs. To demonstrate the OOD properties, we withheld cells exposed to the second to largest dose among all drugs. This choice was made because the vast majority of cells were dead for most of the drugs at the highest dosage, and we would not have enough cells to test the generalization capabilities of the CPA model. Since the latent space representation learned by the CPA is still high‐dimensional, we can use various dimensionality reduction methods to visualize it. We opted for a Kernel PCA computed using a cosine similarity kernel (Appendix Fig S4C). To demonstrate how well CPA captured the dose–response dynamics of individual genes, we looked at the top 2 differentially expressed genes upon all perturbations (Appendix Fig S5). The dose–response curves agree well with the observed data. We evaluate the goodness of the prediction by computing the R2 scores between the means and variances of the predicted gene expression and the real one. We compute this on the entire gene expression vector, and on the top 50 DEGs exclusively for that condition, to make sure that we are capturing the response of genes of interest (Appendix Fig S4B). In order to have a point of reference we formulate a baseline which consists of the R2 scores obtained between the OOD conditions and a random subselection of the training dataset (see Benchmarks section in Materials and Methods). Improvements over this baseline show that the model has learned perturbation and covariate information and has not just modeled an average representation of the training data.

We additionally use the distance of the embedding of an unseen condition from the closest embedding in the observed manifold as a proxy for uncertainty (see Materials and Methods). This distance equals 0 for conditions that were observed during training and increases for points in the perturbation and covariate space that were not presented to the model, for example, dosage values between those sampled in the training dataset (Appendix Fig S4D). This distance increases for combinations of drugs (Appendix Fig S4E), this finding agrees with the fact that the model never saw some drug combinations during training and that such predictions are performed on conditions more distant from those observed during training.

As our third example, we studied the cross‐species dataset from Hagai *et al* (2018). Here we show that the dynamics of the covariate can be a non‐monotonic function, such as time instead of the dose–response. In this example, bone marrow‐derived mononuclear phagocytes from mouse, rat, rabbit, and pig were perturbed with LPS (Appendix Fig S6A). CPA is able to model the response of genes of interest (as indicated in the original study for which the data was generated) over time (Appendix Fig S6B).

### CPA finds interpretable latent spaces in large‐scale single‐cell high‐throughput screens

**Figure 2:**
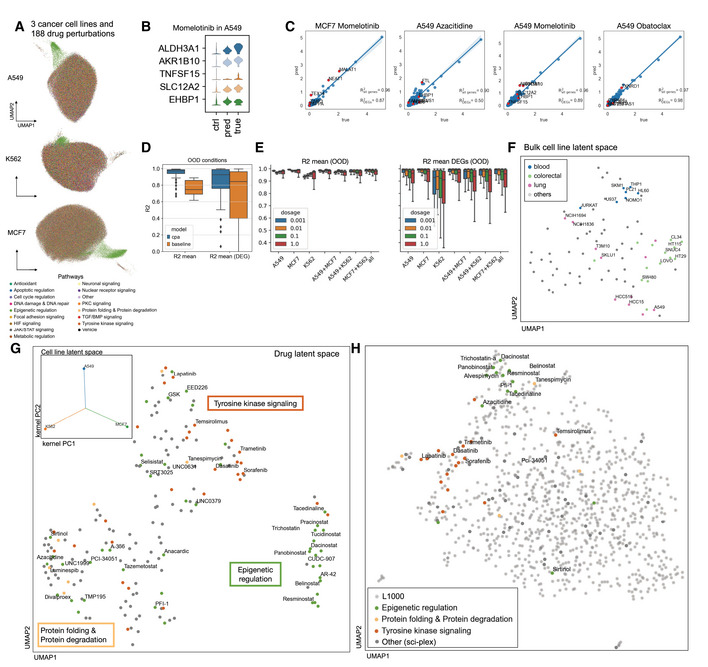
Learning drug and cell line latent representations from massive single‐cell screens of 188 drugs across cancer cell lines UMAP representation of sci‐Plex samples (*n* = 290,889) of A549, K562, and MCF7 cell‐lines colored by pathway targeted by the compounds to which cells were exposed.Distribution of top 5 differentially expressed genes in A549 cells after treatment with Momelotinib, a JAK inhibitor, at the highest dose for real, control and CPA predicted cells.Mean gene expression of 5,000 genes and top 50 DEGs between CPA predicted and real cells together with the top five DEGs highlighted in red for four compounds for which the model did not see any examples of the highest dose.Box plots of $R^{2}$ scores for predicted and real cells for 36 compounds and 108 unique held out conditions across different cell lines. Baseline indicates comparison of each condition with a mean derived from randomly sampled cells.
$R^{2}$ scores box plot for all and top 50 DEGs. Each column represents a scenario where cells exposed with specific dose for all compounds on a cell line or combinations of cell lines were held out from training and later predicted.Latent representation as learned by CPA of 82 cell lines from the L1000 dataset, with some cancer cell lines colored by tissue of origin.Two‐dimensional representation of latent drug embeddings as learned by the CPA. Compounds associated with epigenetic regulation, tyrosine kinase signaling, and protein folding/degradation pathways are colored by their respectively known pathways. The smaller upper right panel shows latent covariate embedding for three cell lines in the data, indicating no specific similarity preference.Latent drug embedding of CPA model trained on the bulk‐RNA cell line profiles of the top 1,000 most tested compounds from the L1000 dataset. Compounds overlapping with the sci‐Plex experiment in (A) are colored according to the same pathway labels as in (G). Data information: Box plots in (E and F) indicate the median (center lines) and interquartile range (hinges), and whiskers represent minimum and maximum values.

The recently proposed sci‐Plex assay (Srivatsan *et al*, 2020) profiles thousands of independent perturbations in a single experiment via nuclear hashing. With this high‐throughput screen, 188 compounds were tested in three cancer cell lines. The panel was chosen to target a diverse range of targets and molecular pathways, covering transcriptional and epigenetic regulators and diverse mechanisms of action. The screened cell lines A549 (lung adenocarcinoma), K562 (chronic myelogenous leukemia), and MCF7 (mammary adenocarcinoma) were exposed to each of these 188 compounds at four doses (10 nM, 100 nM, 1 μM, 10 μM), and scRNA‐seq profiles were generated for altogether 290 thousand cells (Fig 2A). As above, we split the dataset into three subsets: train, test, and OOD. For the OOD case, we held out the highest dose (10 μM) of the 36 drugs with the strongest effect in all three cell lines. Drug, dose, and cell line combinations in the OOD cases were removed from the train and test sets.

Compositional perturbation autoencoder can extrapolate to the unseen OOD conditions with unexpected accuracy, as it captures the difference between control and treated conditions for a compound where it did not see examples with the highest dose. As one example, Momelotinib has a strong differential response to treatment compared to control, as can be seen from the distributions of the top 5 differentially expressed genes (Fig 2B). Despite not seeing the effect of Momelotinib at the highest dose in any of the three cell lines, CPA performs reasonably in inferring the mean and distribution of these genes (Fig 2B). CPA performs well in modeling unseen perturbations, as the correlation of real and predicted values across OOD conditions is overall better than the correlation between target cells and existing cells across different compounds (Fig 2D) when looking at individual conditions (Fig 2C), CPA does well‐recapitulating genes with low and high mean expression in the OOD condition. Furthermore, we compare the performance of CPA against that of scGen on this dataset. scGen is another deep learning method for perturbation prediction in single‐cell datasets. Since this model cannot handle continuous covariates associated with the perturbation, we retain only control and second‐highest dosage cells for this benchmark. We compared the R2 on the predicted means and variances and the Wasserstein distance to measure the whole distribution computed gene‐wise. All metrics were computed on the whole gene vector and also for DEGs. When comparing CPA's predictions with scGen we observed a 1.54% improvement in more straightforward mean prediction compared to higher moments. At the same time, CPA significantly outperformed scGen on variance prediction by 35.85% improvement and similarly outperformed scGen in whole distribution prediction. This benchmark is a very simplified scenario where existing models can be benchmarked and work well on mean prediction but fail to capture whole distribution shifts, which are indeed crucial in single‐cell data since they capture cellular heterogeneity (see Appendix Fig S7).

Compositional perturbation autoencoder performs worse when predicting experiments with more unseen covariates. To assess the ability of the model to generalize to unseen conditions, we trained CPA on 28 splits with different held‐out conditions, with one of the doses held out in anywhere between 1–3 cell lines (Fig 2E). We see here that K562 is the hardest cell line to generalize, when considering training on two cell lines to generalize to another. We also see that extrapolating to the highest dose is a harder task than interpolating intermediate doses, which is consistent with the difficulty of anticipating the experimental effect of a higher dose, versus fitting sigmoidal behavior to model intermediate doses.

After training, CPA learns a compressed representation of the 188 compounds, where each drug is represented by a single 256 dimensional vector (Fig 2G). To test whether the learned drug embeddings are meaningful, we asked if compounds with similar putative mechanisms of action are similar in latent space. This holds for a large set of major mechanisms: we find that epigenetic, tyrosine kinase signaling, and protein formation compounds are clustered together by the model, which suggests the effectiveness of drugs with these mechanisms on these three cancer cell lines which is in line with the findings in the original publication (Srivatsan *et al*, 2020).

We additionally demonstrate that the model learns universal relationships between compounds which remain true across datasets and modalities. Using the same set of compounds tested in the sci‐Plex dataset together with 853 other compounds (for a total of 1,000 compounds), we trained CPA on L1000 bulk perturbation measurement data across 82 cell lines (Musa *et al*, 2019). We observed that CPA works equally well on bulk RNA‐seq data, and also that matched epigenetic and tyrosine kinase signaling compounds present in sci‐Plex were close to each other in the latent representation, suggesting that the learned model similarities apply across datasets (Fig 2H). This holds also for the other learned embeddings: Applying the same similarity metric to the covariate embedding – here the 82 cell lines – we observed that the cell line embedding learned by the model also represents cell line similarity in response to perturbation, as cell lines from blood tissue were clustered together (Fig 2F).

### CPA predicts combinatorial drug effects

**Figure 3:**
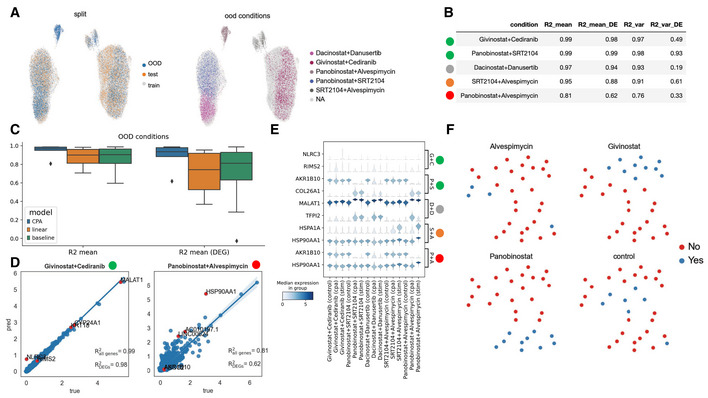
Validation of predictions in new large‐scale drug combination dataset UMAP representation of the combosciplex dataset comprised of *n* = 63,378 cells and 32 perturbation and perturbation combinations in A549 cells. The left UMAP highlights the split used for the following results. The right UMAP shows the five out‐of‐distribution conditions selected and their expression pattern amongst the clusters.The five out‐of‐distribution conditions shown in (A) and model performance per condition. The circles to the left of the condition names indicate a qualitative difficulty assessment of the prediction. Conditions with the green label are dominated by one of the two compounds in the condition and should be relatively easy for the model to predict, while combinations containing Alvespimycin are more transcriptionally dissimilar from conditions seen in training.Benchmark of CPA vs. a linear model vs. the aforementioned random baseline, as measured by R2 on both highly variable genes and the top 50 differentially expressed genes. Box plots indicate the median (center lines) and interquartile range (hinges), and whiskers represents minimum and maximum values.Predicted vs. true, post‐treatment expression values, with the top 5 DEGs colored in red.Violin plots of the top two DEGs per out‐of‐distribution condition and the pre‐stimulation, post‐stimulation, and CPA predicted expression values.UMAP representation of the combination latent vectors learned by CPA. Four individual conditions and the combinations they appear in are highlighted.

We further validated the model trained on the sci‐Plex data by performing a new combination experiment using 13 compounds from the original sci‐Plex in A549 cells. We leveraged the perturbation responses predicted by CPA when trained on sciplex3 (Fig 2) data and selected highly responsive perturbations (which is also reflected in the model's perturbation embedding as separate clusters distinct from control) with which to perform an additional validation experiment. We selected combinations to cover a variety of pathways and response magnitudes, using the second highest dose from the original experiment to capture maximal cell variability. We see that the combinations partitioned themselves into two clusters of behavior (Fig 3A), with the smaller clusters predominantly governed by the transcriptional response to Alvespimycin. We then assessed the ability of the CPA model to predict held out perturbation combinations (Fig 3B). CPA successfully predicted the transcriptional response of compounds which were similar to control, combinations dominated by one compound, and combinations containing the transcriptional response of both compounds (Fig 3B). We can see in Fig 3B that the combination of Panobinostat and Alvespimycin (top left cluster in Fig 3A) was predicted with an R2 of 0.81, despite the model having seen no other cells similar to it as that cluster consisted only of the Panobinostat + Alvespimycin condition and therefore held out entirely. CPA performs better than the control random baseline model described previously and a linear model (see Benchmarks section in Materials and Methods) and accurately predicts the expression levels of highly variable genes (Fig 3C–E). We can then reconstruct the representation between combinations by looking at the latent space derived from combining individual perturbation vectors from the CPA perturbation latent space (Fig 3F). We see that there are three “effect clusters”, represented by Givinostat, Panobinostat, and Alvespimycin in Fig 3F. Givinostat and Panobinostat are both histone deacetylase inhibitors but operate through dissimilar mechanisms of action.

### CPA is an extensible framework for predicting single‐cell perturbations

**Figure 4:**
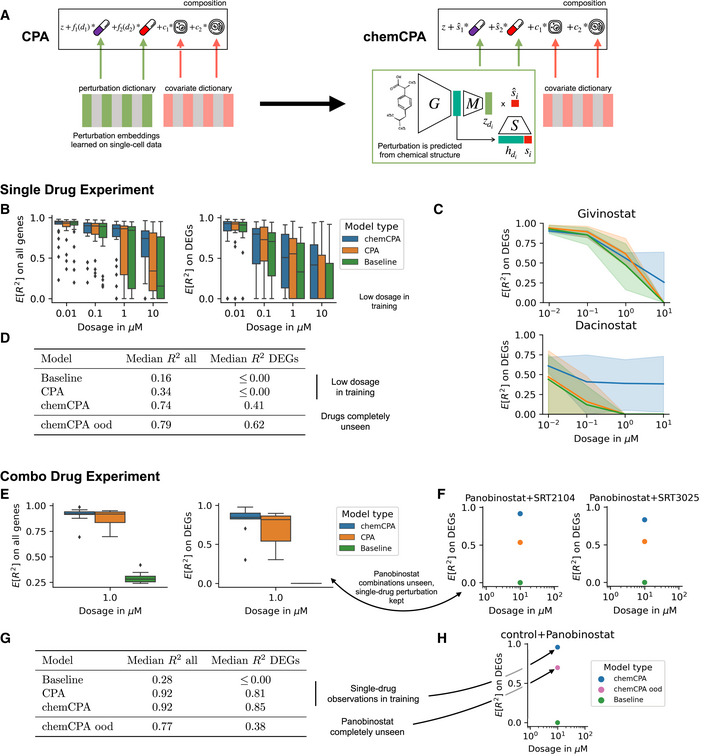
CPA extensibility enables predicting the response to unseen drugs Proposed architecture change for CPA to include chemical prior knowledge. The molecule encoder G maps the chemical information of a compound to a latent drug embedding $h_{d_{i}}$. This module can be based on a pre‐trained graph encoder or molecular fingerprints like here. During training only the perturbation module M and the amortized dosage scaler S are optimized.Comparison between CPA and chemCPA, including a baseline that ignores all drug‐induced perturbation effects. Scores are computed on the sci‐Plex3 (*n* = 290,889) data on a test set that consists of nine compounds. Both the whole genes set (left) and the DEGs (right) are shown.Performance comparison between the models from (B) for two histone deactylation drugs, Givinostat and Dacinostat, across all doses and cell‐lines (shades).Median scores for the models from (B) for the highest dose value of 10 μM, including the result of chemCPA for the setting in which the nine test compounds are completely unseen and excluded from the training and validation.Comparison between CPA and chemCPA on the new combosciplex (*n* = 63,378) including the same type of baseline as in (B). The test set consists of all nine drug combinations that include Panobinostat.Detailed performance comparison between the models from (E) for the two conditions with the highest R2 difference on the DEGs.Median scores for the models from (E), including the result of chemCPA where also the single‐drug Panobinostat observations were held‐out. The median score on the DEGs reduces from 0.85 to 0.38.Showing how well chemCPA is able to predict the single‐drug effect of Panobinostat when it is held out. This is compared to the achieved score when Panobinostat is included in the training set (E). Data information: Box plots indicate the median (center lines) and interquartile range (hinges), and whiskers represent minimum and maximum values.

One of the benefits of the modular architecture employed by CPA is its flexibility and extensibility. While the perturbation dictionary works well for the compositional structure in the latent space, it is limited to the set of compounds present in the training set. Therefore, predicting perturbation responses for compounds not screened in the experiments is not feasible. To enable the prediction of unseen drugs, a recent extension to this, called *chemCPA* (see Materials and Methods), has been proposed by Hetzel *et al* (2022). *chemCPA* introduced a perturbation network that encodes small molecules using their known chemical descriptors (Fig 4A). This perturbation network replaces the perturbation embedding dictionary in CPA. It comprises a pre‐trained molecule encoder *G*, a perturbation encoder *M*, and an amortized dosage scaler *S*. While *M* learns to map from the general chemical embedding to the latent perturbation effect $z_{d_{i}}$ for a compound *d*, the dosage scaler *S* learns to map to the effective dose ${\hat{s}}_{i}$ given the chemical embedding $h_{d_{i}}$ and the applied dosage $s_{i}$.

We applied chemCPA to both the sci‐Plex3 dataset (Fig 2) and the new combination dataset (Fig 3). For the single‐drug prediction experiment, we held out nine compounds (similar to Hetzel *et al*, 2022) as OOD—Dacinostat, Givinostat, Belinostat, Hesperadin, Quisinostat, Alvespimycin, Tanespimycin, TAK‐901, and Flavopiridol. Since CPA's perturbation dictionary is limited to compounds observed in the training set, it is not possible to compare CPA and chemCPA when these drugs are entirely excluded from the training. We kept observations from the two lowest dosages in the training and validation sets to enable comparison within a challenging setting. Hence, excluding the dosage values of 1 μM and 10 μM. The results in this setting illustrate how the chemical prior improved perturbation predictions on both the whole gene set and DEGs across dosages (Fig 4B). Furthermore, we observed that chemCPA generalized particularly well for compounds that belonged to the histone deacetylation pathway (Fig 4C), which is in line with the original sci‐Plex publication (Srivatsan *et al*, 2020) and general support within this perturbation dataset. We report the results from (Hetzel *et al*, 2022) where the OOD drugs were excluded entirely from training and validation (Fig 4D) and observe that the inclusion of the low dosage values diminished chemCPA's performance significantly, demonstrating the importance of high‐dose observation for the training of CPA and chemCPA.

We performed a similar analysis for the combination drug dataset. Here, the OOD set consisted of all nine drug combinations that included Panobinostat and one of SRT3025, PCI‐34051, Sorafenib, Dasatinib, SRT2104, SRT1720, Crizotinib, Alvespimycin, and Curcumin. That is, the single‐drug observation of Panobinostat was kept in the training and validation set, which enabled the comparison of CPA with chemCPA. While both models performed well in this scenario, chemCPA outperformed CPA overall. We should highlight that the clear distribution shift induced by the combination of Alvespimycin and Panobinostat (Fig 3A) could not be fully identified by either model, and both models achieved an $R^{2}$ of about 0.3 on DEGs. In contrast, the other combinations could be predicted with $R^{2}$ higher than 0.6 for the DEGs in the case of the chemCPA model (Fig 4E). The most significant improvement to CPA can be seen in the combination of Panobinostat and SRT2104 (Fig 4F). Through chemCPA's perturbation network, it was possible to exclude the single‐drug perturbation from the training. The results for this OOD setting (Fig 4G) show that in the combination scenario, access to the single‐drug influence is crucial. While the median R2 decreased from 0.85 to 0.38 for the DEGs, the single‐drug perturbation of Panobinostat could still be predicted with high accuracy (Fig 4H).

### CPA allows modeling combinatorial genetic perturbations

Combinatorial drug therapies are hypothesized to address the limited effectiveness of mono‐therapies (Menden *et al*, 2019) and prevent drug resistance in cancer therapies (Jia *et al*, 2009; Menden *et al*, 2019; Adam *et al*, 2020). However, the combined expression of a small number of genes often drives the complexity at the cellular level, leading to the emergence of new properties, behaviors, and diverse cell types (Norman *et al*, 2019). To study such genetic interactions (GIs), recent perturbation scRNA‐seq assays allow us to measure the gene expression response of a cell to the perturbation of genes alone or in combination (Dixit *et al*, 2016; Datlinger *et al*, 2017). While experimental approaches are necessary to assess the effect of combination therapies, in practice, it becomes infeasible to experimentally explore all possible combinations without computational predictions.

**Figure 5:**
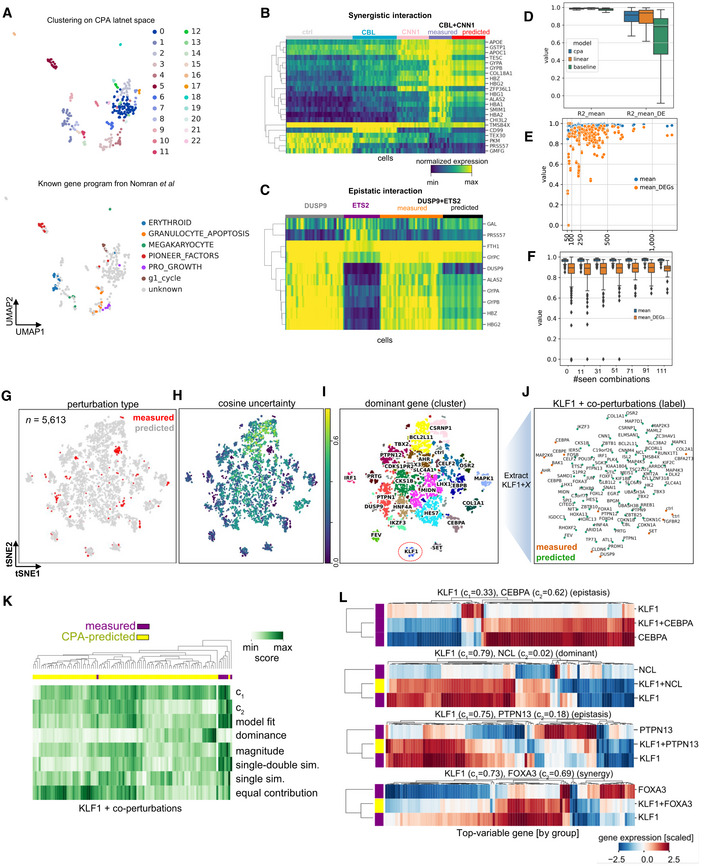
Predicting combinatorial genetic perturbations UMAP inferred latent space using CPA for 281 single and double‐gene perturbations obtained from Perturb‐seq (*n* = 108,497). Each dot represents a genetic perturbation. Coloring indicates known gene programs associated with perturbed genes from the original publications.Measured and CPA‐predicted gene expression for cells linked to a synergistic gene pair (*CBL + CNN1*). Gene names taken from the original publication.As (b) for an epistatic (*DUSP9 + ETS*) gene pair. Top 10 DEGs of *DUSP9 + ETS* co‐perturbed cells versus control cells are shown.R2 values of mean gene‐expression of measured and predicted cells for all genes (black) or top 100 DEGs for the prediction of all 131 combinations for CPA, linear model, and the baseline (13 trained models, with ≈10 tested combinations each time).R2 values of predicted and real mean gene‐expression versus number of cells in the real data.R2 values for predicted and real cells versus number of combinations seen during training.UMAP of mean gene expression in each measured (*n* = 284, red dots) and CPA‐predicted (*n* = 5,329, gray dots) perturbation combinations.As (g), showing measurement uncertainty (cosine similarity).As (g), showing dominant genes in Leiden clusters (25 or more observations). KLF1 cluster is highlighted with red dotted circle.UMAP of mean expression of cells with KLF1 as a common co‐perturbed gene.Hierarchical clustering of linear regression associated metrics between *KLF1* with co‐perturbed genes, in measured and predicted cells. Each row indicates summary parameters obtained when fitting a linear regression for double predictions using single perturbations as predicting variables, and relationships between coefficients c_1_ and c_2_. For definitions see Materials and Methods.Scaled gene expression changes (versus control) of RF‐selected genes (x‐axis) in measured (purple) and predicted (yellow) perturbations (y‐axis). Headers indicate gene‐wise regression coefficients, and interaction mode suggested by (Norman *et al*, 2019). Data information: Box plots indicate the median (center lines) and interquartile range (hinges), and whiskers represent minimum and maximum values.

To pursue this aim, we applied our CPA model to scRNA‐seq data collected from Perturb‐seq (single‐cell RNA‐sequencing pooled CRISPR screens) to assess how overexpression of single or combinatorial interactions of 105 genes (i.e., single gene x, single gene y, and pair x + y) affected the growth of K562 cells (Norman *et al*, 2019). In total, this dataset contains 284 conditions measured across ≈108,000 single‐cells, where 131 are unique combination pairs (i.e., x + y) and the rest are single gene perturbations or control cells. We observed that the latent genetic interaction manifold placed GIs inducing known and similar gene programs close to each other (Fig 5A). We further compared our latent space clustering to mean gene expression embedding similar to the original publication (Appendix Fig S8). Overall, CPA latent space grouped similar perturbation as the mean gene expression embedding achieving more granular clusters leading to better clustering metrics. Next, we sought to assess our ability to predict specific genetic interactions. We examined a synergistic interaction between *CBL* and *CNN1* in driving erythroid differentiation which has been previously validated (Norman *et al*, 2019). We trained a CPA model with *CBL + CNN1* held out from the training data. Overexpression of either gene leads to the progression of cells from control to single perturbed and doubly perturbed cells (Appendix Fig S9A) toward the erythroid gene program. Overexpression of both *CBL* and *CNN1* up‐regulate known gene markers (Norman *et al*, 2019) such as hemoglobins (see *HBA1/2* and *HBG1/2* in Fig 5B). We observed that our model successfully predicted this synergistic interaction, recapitulating patterns similar to real data and inline with the original findings (Fig 5C). We further evaluated CPA to predict a previously reported (Norman *et al*, 2019) genetic epistatic interaction between *DUSP9* and *ETS1*, leading to domination of the *DUSP9* phenotype in doubly perturbed cells (Fig 5C).

To systematically evaluate the CPA's generalization behavior, we trained 13 different models while leaving out all cells from ≈10 unique combinations covering all 131 doubly perturbed conditions in the dataset, which were predicted following training. We compared CPA predictions with linear and random baselines described in the previous section (Fig 5D). Surprisingly, The baseline approach achieved accurate predictions on par with both CPA and linear models. This observation demonstrates that the transcriptomic effects caused by different perturbations are very similar and the dataset contains limited non‐linear effects on the whole transcriptome level. Thus, a linear model or simple baseline can provide accurate predictions on mean as opposed to combinatorial drug effects previously demonstrated. The reported R2 values showed robust prediction for most of the perturbations: lower scores were seen for perturbations where the evaluation was noisy due to sample scarcity (*n* < 100), or when one of the perturbations was only available as singly perturbed cells in the data, leading the model to fail to predict the unseen combination (Fig 5E, see Appendix Figs S9 and S10). To further understand when CPA performance deteriorated, we first trained it on a subset with no combinations seen during training, and then gradually increased the number of combinations seen during training. We found that overall prediction accuracy improved when the model was trained with more combinations and that it could fail to predict DEGs when trained with fewer combinations (see *n* < 71 combinations in Fig 5F).

Hence, once trained with sufficiently large and diverse training data, CPA could robustly predict unseen perturbations. We next asked whether our model could generalize beyond the measured combinations and generate *in silico* all 5,329 combinations, which were not measured in the real dataset, but made up ≈98% of all possibilities. To study the quality of these predictions, we trained a model where all combinations were seen during training to achieve maximum training data and sample diversity. We then predicted 50 single‐cells for all missing combinations. We embedded (Fig 5G) mean gene expression vector of all measured and generated data while reporting an uncertainty value for each condition. (Fig 5H). We hypothesized that the closer the generated embedding was to the measured data. the more likely it was to explore a similar space of the genetic manifold around the measured data. Equipped with this information, we annotated the MIS clusters based on gene prevalence, finding that single genes (i.e. gene x) paired with other genes (i.e., y) as combinations (i.e., x + y) are a main driver of cluster separation (Fig 5I). Genes without measured double perturbations were less likely to be separated as independent clusters using the newly predicted transcriptomic expression (Appendix Fig S11A), suggesting that their interaction‐specific effects were less variable than cases with at least one double perturbation available in the training data. Meanwhile, points distant from measured data can potentially indicate novel gene‐interaction behaviors such as in the case of *KLF1* with co‐perturbed genes (Fig 5J). However, this would require additional consideration and validation steps.

To investigate the type of interaction between the newly predicted conditions, we compared the differences between double and single perturbations versus control cells and thus annotated their interaction modes adapted from the original publication (Norman *et al*, 2019). In each gene‐specific cluster, we observed variability across these values, suggesting that our predictions contained granularity that went beyond single gene perturbation effects and could not be fully dissected by two‐dimensional embeddings. Upon curation of gene perturbations using these metrics and the levels of experimental data available (Appendix Fig S11B), we decided to predict and annotate interaction modes based on these values when double measurements were available for at least one gene. For example, we observed clustering of *KLF1* and partner gene perturbation pairs solely from these metrics in most of the measured data points, suggesting the existence of several interaction modes that cannot be fully described with bidimensional embeddings (Fig 5K). Extraction of gene‐pairs based on these values and their variability allowed us to extract interaction types and label those into interpretable categories, via thresholding as previously proposed by Norman *et al* When we further examine the differentially expressed genes in each co‐perturbation, these metrics validated previously reported epistatic interactions (*CEBPA*), and proposed new ones with *KLF1*‐dominant behavior (*NCL*), gene synergy (*FOXA3*), and epistasis (*PTPN13*), among others (Fig 5L). Repeating this analysis across all measured and predicted double perturbations, we found genes with potential interaction prevalences (Appendix Fig S11C). Among genes which repeatedly respond to several perturbations, we found common gene expression trends in both direction and magnitude (Appendix Fig S11D), suggesting that variation is modulated by conserved gene regulatory principles that are potentially captured in our learned model.

Altogether, our analysis indicated that double perturbation measurements can be generated by CPA by leveraging genetic perturbation data, which when combined with an uncertainty metric allows us to generate and interpret gene regulatory rules in the predicted gene–gene perturbations.

## Discussion

*In silico* prediction of cell behavior in response to a perturbation is critical for optimal experiment design and the identification of effective drugs and treatments. With CPA, we have introduced a versatile and interpretable approach to modeling cell behaviors at single‐cell resolution. CPA is implemented as a neural network trained using stochastic gradient descent, scaling up to millions of cells and thousands of genes.

We applied CPA to a variety of datasets and tasks, from predicting single‐cell responses to learning embeddings, as well as reconstructing the expression response of compounds, with variable drug‐dose combinations. Specifically, we illustrated the modeling of perturbations across dosage levels and time series, and have demonstrated applications in drug perturbation studies, as well as genetic perturbation assays with multiple gene knockouts, revealing potential gene–gene interaction modes inferred by our model predicted values. CPA combines the interpretability of linear decomposition models with the flexibility of nonlinear embedding models.

While CPA performed well in our experiments, it is well known that in machine learning there is no free lunch, and as with any other machine learning model, CPA will fail if the test data are very different from the training data. To alert CPA users to these cases, it is crucial to quantify model uncertainty. To do so, we implemented a distance‐based uncertainty score to evaluate our predictions. The current heuristic for uncertainty estimation originates from the compositional formulation in CPA. Such formulation results in very different covariate/perturbation vector combinations to those observed in training data will have a higher distance and thus higher uncertainty than covariate/perturbation observed in training (see Appendix Figs S4 and S12). Yet, like other in silico predictive models, CPA's predictions cannot replace experimental validation; instead, it can serve as a potential guide to efficiently conduct experiments. Additionally, scalable Bayesian uncertainty models are promising alternatives for future work (Gal & Ghahramani, 2016). Although we opted to implement a deterministic autoencoder scheme, extensions toward variational models (Lopez *et al*, 2018; Lotfollahi *et al*, 2020), as well as cost functions other than mean squared error (Eraslan *et al*, 2019) are straightforward. In addition to model uncertainty, the data uncertainty and biases play an important role in biasing the model toward specific prediction regimes. For example, genes co‐measured with other genes tend to show more complex predictions than the ones where either one or neither gene was co‐measured in combinations, in which case the model tends to predict single‐gene effects (dominance) (see Appendix Fig S11D). The nature of these multivariate biases and whether it is solely data‐driven or linked to Biology should be the focus of additional work. We hope this insight contributes to the design of future combinatorial screenings. Finally, we leveraged one fixed architecture with very similar hyperparameters. However, to achieve the best performance, systematic hyperparameter sweeping is needed; for example, in cases where many perturbations and covariates are present different weights for disentanglement loss might be required to ensure the basal state is free of perturbation and covariate information.

Aside from CPA, existing methods (Lotfollahi *et al*, 2020; Russkikh *et al*, 2020) such as scGen (Lotfollahi *et al*, 2019) have also been shown capable of predicting single‐cell perturbation responses when the dataset contains no combinatorial treatment or dose‐dependent perturbations. Therefore, it may be beneficial to benchmark CPA against such methods in less complicated scenarios with few perturbations. However, this approach might not be practical, considering the current trend toward the generation of massive perturbation studies (Dixit *et al*, 2016; Norman *et al*, 2019; Srivatsan *et al*, 2020).

Currently, the model is based on gene expression alone, so it cannot directly capture other levels of interactions or effects, such as those due to post‐transcriptional modification, signaling, or cell communication. However, due to the flexibility of neural network‐based approaches, CPA could be extended to include other modalities, for example via multimodal single‐cell CRISPR (Frangieh *et al*, 2021; Papalexi *et al*, 2021) combined scRNA‐seq and ATAC‐seq (Clark *et al*, 2018; Chen *et al*, 2019) and CUT&Tag (Kaya‐Okur *et al*, 2019; Wu *et al*, 2021). In particular, we expect spatial transcriptomics (Rodriques *et al*, 2019; van den Brink *et al*, 2020) to be a valuable source for extensions to CPA due to its high sample number and the dominance of DL models in computer vision.

The CPA model is not limited to single‐cell perturbations. While we chose the single‐cell setting due to the high sample numbers available, the CPA could readily be applied to large‐scale bulk cohorts, in which covariates might be patient ID or transcription factor perturbation. These and any other available attributes could be controlled independently (Lample *et al*, 2017) to achieve compositional, interpretable predictions. Any bulk compositional model may be combined with a smaller‐scale single‐cell model to compose truly multi‐scale models of observed variance. The flexibility of the DL setting will also allow addition of constraints on perturbation or covariate latent spaces. These could, for example, be the similarity of chemical compounds (Mater & Coote, 2019), or clinical‐covariate induced differences of patient IDs. The key feature of the CPA versus a normal autoencoder is its latent space disentanglement and the induced interpretability of the perturbations in the context of cell states and covariates. Eventually, any aim in biology is not only blind prediction, but mechanistic understanding. This objective is exemplified by the direction that DL models are taking in sequence genomics, where the aim is not only the prediction of new interactions, but also the interpretation of the learned gene regulation code. We therefore believe that CPA can not only be used as a hypothesis generation tool for *in silico* screens but also as an overall data approximation model. Deviations from our assumed data generation process (see Materials and Methods) would then tell us about missing information in the given data set and/or missing aspects in the factor model. By including multiple layers of regulation, the resulting model can grow in flexibility for prediction and for mechanistic understanding of for example synergistic gene regulation or other interactions.

Finally, we expect CPA to facilitate new opportunities in expression‐based perturbation screening, not only to learn optimal drug combinations, but also in how to personalize experiments and treatments by tailoring them based on individual cell response.

## Materials and Methods

### Reagents and tools table

**Table:** 

Reagent/Resource	Reference or Source	Identifier or Catalog Number
*python v3.7*	https://www.python.org/	
*pytorch v1.10*	https://pytorch.org/	
*scanpy v1.8*	https://pypi.org/project/scanpy/	
*anndata v0.7*	https://pypi.org/project/anndata/	

### Methods and Protocols

#### Data generating process

We consider a dataset $D={\left\{\left(x_{i},d_{i},c_{i}\right)\right\}}_{i=1}^{N}$, where each $x_{i}\in R^{G}$ describes the gene expression of G genes from cell i. The perturbation vector $d_{i}=\left(d_{i,1},\ldots ,d_{i,M}\right)$ contains elements $d_{i,j}\geq 0$ describing the dose of drug *j* applied to cell *i*. If $d_{i,j}=0$, this means that perturbation *j* was not applied to cell *i*. Unless stated otherwise, the sequel assumes column vectors. Similarly, the vector of vectors $c_{i}=\left(c_{i,1},\ldots c_{i,K}\right)$ contains additional discrete covariates such as cell‐types or species, where each covariate is itself a vector. More specifically, $c_{i,j}$ is a *K*_*j*_‐dimensional one‐hot vector.

We assume that an unknown generative model produced our dataset *D*. The three initial components of this generative process are a latent (unobserved) basal latent state $z_{i}^{\text{basal}}$ for cell i, together with its perturbation vector $d_{i}$ and covariate vector $c_{i}$. We assume that the basal latent state is independent from the perturbation vector $d_{i}$ and covariate vector $c_{i}$. Next, we form the latent (also unobserved) perturbed latent state $z_{i}$ as:

(1) $$z_{i}=z_{i}^{\text{basal}}+V^{\text{perturbation}}\cdot \left(f_{1}\left(d_{i,1}\right),\ldots ,f_{M}\left(d_{i,M}\right)\right)+\sum_{j=1,\ldots ,K}V^{{\mathrm{cov}}_{j}}\cdot c_{i,j}c$$

In this equation, each column of the matrix $V^{\text{perturbation}}\in R^{d\times M}$ represents a d‐dimensional embedding for one of the M possible perturbations represented in $d_{i}$. Similarly, each column of the matrix $V^{{\mathrm{cov}}_{j}}\in R^{d\times K_{j}}$ represents a d‐dimensional embedding for the j‐th discrete covariate, represented as a $K_{j}$‐dimensional one‐hot vector $c_{i,j}$. The functions $f_{j}:R\to R$ scale non‐linearly each of the $d_{i,j}$ in the perturbation vector $d_{i}$, therefore implementing M independent dose–response (or time‐response) curves. Finally, we assume that the generative process returns the observed gene expression $x_{i}$ by means of an unknown decoding distribution $p\left(x_{i}|z_{i}\right)$. This process builds the observation $\left(x_{i},d_{i},c_{i}\right)$, which is then included in our dataset D.

#### Compositional perturbation autoencoder (CPA)

Assuming the generative process described above, our goal is to train a machine learning model $x_{i}^{\prime }=M\left(\left(x_{i},d_{i},c_{i}\right),d^{\prime }\right)$ such that, given a dataset triplet $\left(x_{i},d_{i},c_{i}\right)$ as well as a target perturbation $d^{\prime }$, estimates the gene expression $x_{i}^{\prime }$. The term $x_{i}^{\prime }$ represents what would the counterfactual distribution of the gene expression $x_{i}$ with covariates $c_{i}$ look like, had it been perturbed with $d^{\prime }$ instead of $d_{i}$.

Given a dataset and a learning goal, we are now ready to describe our proposed model, the CPA. In the following, we describe separately how to train and test CPA models.

#### Training

The training of a CPA model consists in auto‐encoding dataset triplets $\left(x_{i},d_{i},c_{i}\right)$. That is, during training, a CPA model does not attempt to answer counterfactual questions. Instead, the training process consists in (i) encoding the gene expression $x_{i}$ into an estimated basal state ${\hat{z}}_{i}^{\text{basal}}$ that does not contain any information about $\left(d_{i},c_{i}\right)$, (ii) combining ${\hat{z}}_{i}^{\text{basal}}$ with learnable embeddings about $\left(d_{i},c_{i}\right)$ to form an estimated perturbed state ${\hat{z}}_{i}$, and (iii) decoding ${\hat{z}}_{i}$ back into the observed gene expression $x_{i}$.

More specifically, following a forward pass of the CPA model first encodes the observed gene expression $x_{i}$ into an estimated basal state:

$${\hat{z}}_{i}^{\text{basal}}={\hat{f}}^{\mathrm{enc}}\left(x_{i}\right).$$

The estimated basal state is used as input to the auxiliary classifiers (see next paragraph) and also to compute the estimated perturbed state ${\hat{z}}_{i}$:

(2) $${\hat{z}}_{i}\;≔\;{\hat{z}}_{i}^{\text{basal}}+{\hat{V}}^{\text{perturbation}}\cdot \left({\hat{f}}_{1}\left(d_{i,1}\right),\ldots {\hat{f}}_{M}\left(d_{i,M}\right)\right)+\sum_{j=1,\ldots ,K}{\hat{V}}^{{\mathrm{cov}}_{j}}\cdot c_{i,j}$$

Contrary to (1), this expression introduces three additional learnable components: the perturbation embeddings ${\hat{V}}^{\text{perturbation}}$, the covariate embeddings ${\hat{V}}^{\mathrm{cov}}$ and the learnable dose–response curves $\left({\hat{f}}_{1},\ldots ,{\hat{f}}_{M}\right)$, here implemented as small neural networks constrained to satisfy ${\hat{f}}_{j}\left(0\right)=0$.

As a final step, a decoder ${\hat{f}}^{\mathrm{dec}}$ accepts the estimated perturbed state ${\hat{z}}_{i}$ and returns ${\hat{f}}_{\mu }^{\mathrm{dec}}\left({\hat{z}}_{i}\right)$ and ${\hat{f}}_{\sigma^{2}}^{\mathrm{dec}}\left({\hat{z}}_{i}\right)$, that is, the estimated mean and variance of the counterfactual gene expression $x_{i}^{\prime }$.

To train CPA models, we use three loss functions. First, the reconstruction loss function is the Gaussian negative log‐likelihood:

(3) $$l_{i}\;≔\frac{\text{logs}\left({\hat{f}}_{\sigma^{2}}^{\mathrm{dec}}\left({\hat{z}}_{i}\right)\right)}{2}+\frac{{\left({\hat{f}}_{\mu }^{\mathrm{dec}}\left({\hat{z}}_{i}\right)-x_{i}^{\prime }\right)}^{2}}{2\cdot s\left({\hat{f}}_{\sigma^{2}}^{\mathrm{dec}}\left({\hat{z}}_{i}\right)\right)},$$

where $s\left(\sigma^{2}\right)=\log\left(\exp\left(\sigma^{2}+10^{-3}\right)+1\right)$ enforces a positivity constraint on the variance and adds numerical stability. This loss function rewards the end‐to‐end auto‐encoding process if producing the observed gene expression $x_{i}$.

Second, and according to our assumptions about the data generating process, we are interested in removing the information about $\left(d_{i},c_{i}\right)$ from ${\hat{z}}_{i}^{\text{basal}}$. To achieve this information removal, we follow an adversarial approach (Lample *et al*, 2017). In particular, we set up the following auxiliary loss functions:

$$l_{i}^{d}\;≔\;\text{CrossEntropy}\left({\hat{f}}_{d}^{\mathrm{adv}}\left({\hat{z}}_{i}^{\text{basal}}\right),d_{i}\right),l_{i,j}^{c}≔\text{CrossEntropy}\left({\hat{f}}_{c_{i,j}}^{\mathrm{adv}}\left({\hat{z}}_{i}^{\text{basal}}\right),c_{i,j}\right),\;\forall j=1,\ldots ,K.$$

The functions ${\hat{f}}_{d}^{\mathrm{adv}}$, ${\hat{f}}_{c_{i,j}}^{\mathrm{adv}}$ are a collection of neural network classifiers trying to predict $\left(d_{i},c_{i}\right)$ given the estimated basal state ${\hat{z}}_{i}^{\text{basal}}$.

Given this collection of losses, the training process is an optimization problem that repeats the following two steps:

1. sample $\left(x_{i},d_{i},c_{i}\right)∼D$, minimize $l_{i}^{d}+\sum_{j}l_{i,j}^{c}$ by updating the parameters of ${\hat{f}}_{d}^{\mathrm{adv}}$ and ${\hat{f}}_{c_{i,j}}^{\mathrm{adv}}$, for all j=1,…,K;
2. sample $\left(x_{i},d_{i},c_{i}\right)∼D$, minimize $l_{i}-\lambda \cdot \left(l_{i}^{d}+\sum_{j}l_{i,j}^{c}\right)$ by updating the parameters of the encoder ${\hat{f}}^{\mathrm{enc}}$, the decoder ${\hat{f}}^{\mathrm{dec}}$, the perturbation embeddings ${\hat{V}}^{\text{perturbation}}$, the covariate embeddings ${\hat{V}}^{{\mathrm{cov}}_{j}}$ for all j=1,…,K, and the dose–response curve estimators $\left({\hat{f}}_{1},\ldots ,{\hat{f}}_{M}\right)$.

#### Testing

Given an observation $\left(x_{i},d_{i},c_{i}\right)$ and a counterfactual treatment $d^{\prime }$, we can use a trained CPA model to answer what would the counterfactual distribution of the gene expression $x_{i}$ with covariates $c_{i}$ look like, had it been perturbed with $d^{\prime }$ instead of $d_{i}$. To this end, we follow the following process:

1. Compute the estimated basal state ${\hat{z}}_{i}^{\text{basal}}={\hat{f}}^{\mathrm{enc}}\left(x_{i}\right)$;
2. Compute the counterfactual perturbed state ${\hat{z}}_{i}^{\prime }$
$${\hat{z}}_{i}^{\prime }\;≔\;{\hat{z}}_{i}^{\text{basal}}+{\hat{V}}^{\text{perturbation}}\cdot \left({\hat{f}}_{1}\left(d\prime_{i,1}\right),\ldots {\hat{f}}_{M}\left(d\prime_{i,M}\right)\right)+\sum_{j=1,\ldots ,K}{\hat{V}}^{{\mathrm{cov}}_{j}}\cdot c_{i,j}.$$

Note that in the previous expression, we are using the counterfactual treatment $d^{\prime }$ instead of the observed treatment $d_{i}$.

3 Compute and return the counterfactual gene expression mean $x_{i,\mu }^{\prime }$:
$$x_{i,\mu }^{\prime }={\hat{f}}_{\mu }^{\mathrm{dec}}\left({\hat{z}}_{i}^{\prime }\right),$$
  and variance $x_{i,\sigma^{2}}^{\prime }$:
$$x_{i,\sigma^{2}}^{\prime }={\hat{f}}_{\sigma^{2}}^{\mathrm{dec}}\left({\hat{z}}_{i}^{\prime }\right).$$

#### Hyper‐parameters and training

For each dataset, we perform a random hyper‐parameter search of 100 trials. Table 1 outlines the distribution of values for each of the hyper‐parameters involved in CPA training.

**Table 1:** Hyperparameter selection.

Group	Hyperparameter	Default value	Random search distribution
General	Embedding dimension	256	RandomChoice([128, 256, 512])
	Batch size	128	RandomChoice([64, 128, 256, 512])
	Learning rate decay, in epochs	45	RandomChoice([15, 25, 45])
Nonlinear scalers	Hidden neurons, nonlinear scalers	64	RandomChoice([32, 64, 128])
	Hidden layers	2	RandomChoice([1, 2, 3])
	Learning rate	1e‐3	10^Uniform (−4, −2)^
	Weight decay	1e‐7	10^Uniform (−8, −5)^
Encoder and decoder	Hidden neurons, encoder, and decoder	512	RandomChoice([256, 512, 1,024])
	Hidden layers	4	RandomChoice([3, 4, 5])
	Learning rate	1e‐3	10^Uniform (−4, −2)^
	Weight decay	1e‐6	10^Uniform (−8, −4)^
Discriminator	Hidden neurons, discriminator	128	RandomChoice([64, 128, 256])
	Hidden layers	3	RandomChoice([2, 3, 4])
	Regularization strength	5	10^Uniform (−2, 2)^
	Gradient penalty	3	10^Uniform (−2, 1)^
	Learning rate	3e‐4	10^Uniform (−5, −3)^
	Weight decay	1e‐4	10^Uniform (−6, −3)^
	Number of learning steps	3	RandomChoice([1, 2, 3, 4, 5])

#### Model evaluation

We use several metrics to evaluate the performance of our model: (i) quality of reconstruction for in and OOD cases and (ii) quality of disentanglement of cell information from perturbation information. We split each dataset into three subsets: train, validation, and OOD. For OOD cases, we choose combinations of perturbations that exhibit unseen behavior. This usually corresponds to the most extreme drug dosages. We select one perturbation combination as “control”. Usually, these are Vehicle or DMSO if real control samples are present in the dataset; otherwise, we choose a drug perturbation at a lower dosage as “control”. For the evaluation, we use the mean squared error of the reconstruction of an individual cell and average it over the cells for the perturbation of interest. As an additional metric, we use classification accuracy in order to check how well the information about the drugs was separated from the information about the cells.

#### chemCPA

The chemCPA model (Hetzel *et al*, 2022) extends CPA by a perturbation network that replaces CPA's perturbation dictionary with a neural network that maps chemical information to the latent drug encoding. This extension allows to infer latent perturbation embedding for compounds that are not originally present in the dataset, i.e. predicting cellular perturbation effects for drugs that are completely unseen. For both presented experiments, we use the default training scripts of the chemCPA model as provided by Hetzel *et al* (2022) and available at (https://github.com/theislab/chemCPA). For a fair comparison, we tested the same set of hyperparameters for CPA and chemCPA. As molecule encoder G, we rely on the molecular fingerprints, computed with RDKit (Landrum, 2006), as these have shown competitive performance in the performed benchmark in (Hetzel *et al*, 2022). In the combination setting, we compute one forward pass of the perturbation network per compound and perform latent space arithmetic identically to CPA.

#### Benchmarks

##### Random baseline

We call random baseline the R2 between means and variances of gene expression between a certain condition (e.g., OOD) and a random subset of the training data. This gives an idea of how heterogeneous gene expression is in the dataset. Improvements over this baseline mean that the model has learned meaningful information regarding covariates and perturbations and has not naively learned a mean representation of the training data.

##### Linear baseline

As a baseline for combinatorial perturbations we use the average between the pseudobulked gene expressions of the two perturbations.

$${\tilde{x}}_{\mathrm{AB}}=\frac{x_{A}^{-}+x_{B}^{-}}{2}$$

##### scGen

We used scGen (Lotfollahi *et al*, 2019) with default parameters as according to the tutorial described here.

##### Uncertainty estimation

To estimate the uncertainty of the predictions we use as a proxy the minimum distance between the queried perturbation and the set of conditions (covariate + perturbation combinations) seen during training (Appendix Fig S12). Intuitively, we expect predictions on queried conditions that are more distant from the set of seen conditions to be more uncertain. To estimate this distance we first compute the set of embeddings of the training covariate and perturbation combinations:

$${\hat{z}}^{\text{comb}}={\hat{V}}^{\text{perturbation}}\cdot \left({\hat{f}}_{1}\left(d\prime_{1}\right),\ldots {\hat{f}}_{M}\left(d\prime_{M}\right)\right)+\sum_{j=1,\ldots ,K}{\hat{V}}^{{\mathrm{cov}}_{j}}\cdot c_{j}.$$

The latent vector for the queried condition is obtained in the same manner. The cosine and euclidean distances from the training embedding set are computed and the minimum distance is used as a proxy for uncertainty. Our perturbation and covariate embeddings are not normalized. Therefore, cosine and euclidean distances yield different interpretations and orderings. With the cosine distance, we measure the distance between the vectors' orientation, assuming their magnitude is not essential. In contrast, the euclidean distance measures the distance between the two vectors considering their magnitude.

$$u_{\text{cosine}}=\min\left(1-S_{C}\left({\hat{z}}^{\text{query}},{\hat{z}}^{\text{comb}}\right)\right)\;u_{\text{eucl}}=\min\left(d\left({\hat{z}}^{\text{query}},{\hat{z}}^{\text{comb}}\right)\right)$$

where $S_{C}\left(x,y\right)$ stands for the cosine similarity and $d\left(x,y\right)$ for the euclidean distance between the two vectors.

With this methodology, in the case of a drug screening experiment, if we query a combination of cell type, drug, and dosage that was seen during training, we get an uncertainty of zero since this combination was present in the training set. It is important to note that with this method, we obtain a condition‐level uncertainty in that all cells predicted under the same query will have the same uncertainty, thus not considering cell‐specific information.

##### R2 score

We used the *r2_score* function from *scikit‐learn*, which reports the R2 (coefficient of determination) regression score.

##### Clustering metrics

We used *silhouette_score* and *homogeneity_score* functions from *scikit‐learn* to calculate metrics. Original labels from Norman *et al* were used to assess cluster homogeneity. The cluster homogeneity is high when all clusters contain only data points that are members of a single cluster. We tried multiple values of K (k in [3,4,5,6,8,7,8,9,10]) to construct the neighborhood graph and also multiple resolutions (from low to high resolution) for Leiden clustering (resolution in [0.3, 0.4, 0.5, 0.6, 0.7,0.75, 0.8,0.85, 0.9, 1]) to maximize the normalized_mutual_info_score() from *scikit‐learn* between clusters and the ground truth gene programs labels obtained from the original study. The best result across multiple hyperparameters for each method was selected and compared.

#### Datasets

##### Kang *et al*

The dataset was obtained from Stuart *et al* (2019) tutorial. The object includes PBMCs from eight patients with Lupus. The cells are either treated with IFN‐*β* or control cells (Kang *et al*, 2018). We then proceeded with normalization, log (*x* + 1)‐transformation and the selection of 5,000 HVGs using SCANPY.

##### Genetic CRISPR screening experiment

We obtained the raw count matrices from Norman *et al* (2019) from GEO (accession ID GSE133344). According to authors guide, we excluded “NegCtrl1_NegCtrl0__NegCtrl1_NegCtrl0” control cells and merged all unperturbed cells as one “ctrl” condition. We then normalized and log‐transformed the data using SCANPY and selected 5,000 HVGs for training. The processed dataset contained 108,497 cells.

##### Cross‐species experiment

The data was generated by Hagai *et al* (2018) and downloaded from ArrayExpress (accession: E‐MTAB‐6754). The data consists of 119,819 phagocytes obtained from four different species: mouse, rat, pig, and rabbit. Phagocytes were treated with lipopolysaccharide (LPS) and the samples were collected at different time points: 0 (control), 2, 4, and 6 h after the beginning of treatment. All genes from non‐mouse data were mapped to the respective orthologs in the mouse genome using Ensembl ID annotations. We filtered out cells with a percentage of counts belonging to mitochondrial genes higher than 20%, then proceeded to normalize and log‐transform the count data. For training and evaluation, we selected 5,000 HVG using SCANPY. After filtering, the data consists of 113,400 cells.

##### sci‐Plex 2

The data was generated by Srivatsan *et al* (2020) and downloaded from GEO (GSM4150377). The dataset consists of A549 cells treated with one of the following four compounds: dexamethasone, Nutlin‐3a, BMS‐345541, or Vorinostat (SAHA). The treatment lasted 24 h across seven different doses. The count matrix obtained from GEO consists of 24,262 cells. During QC we filtered cells with fewer than 500 counts and 720 detected genes. We discarded cells with a percentage of mitochondrial gene counts higher than 10%, thus reducing the dataset to 14,811 cells. Genes present in fewer than 100 cells were discarded. We normalized the data using the size factors provided by the authors and log‐transformed it. We selected 5,000 HVGs for training and further evaluations.

##### sci‐Plex 3

The data was generated by Srivatsan *et al* (2020) and downloaded from GEO (GSM4150378). The dataset consists of three cancer cell lines (A549, MCF7, K562), which are treated with 188 different compounds with different mechanisms of action. The cells are treated with 4 dosages (10, 100, 1,000, and 10,000 nM) plus vehicle. The count matrix obtained from GEO consists of 581,777 cells. The data was subset to half its size, reducing it to 290,888 cells. We then proceeded with log‐transformation and the selection of 5,000 HVGs using SCANPY.

##### Combosciplex

###### Experiment details

Drug dose combinations were administered as described previously (Srivatsan *et al*, 2020). Briefly, A549 cells were grown in DMEM media (ThermoFisher Scientific; cat no. 11966025) supplemented with 10% FBS and 1% Penicillin–Streptomycin. These cells were then seeded at 25,000 cells/well in a 96‐well flat bottom plate (ThermoFisher Scientific, cat no. 12‐656‐66). Prior to treatment, stock compounds from SelleckChem (stock concentration of 10 mM) were first diluted to 1:10 in DMSO, followed by a 1:10 dilution in PBS. From this dilution, 1 μl of each compound was added to a culture well in a 96‐well plate containing 99 μl of media. Treatments were performed one day after plating and resulted in a final concentration of 1 μM for each compound. After 24 h of growth at 37°C and 5% CO_2_, media was removed, cells were washed once with cold PBS, and then enzymatically detached using 50 μl of TrypLE at 37°C for 5 min (ThermoFisher; cat no. 12604013). After detachment, cells were quenched with 100 μl of media containing FBS, transferred to a 96‐well V‐bottom plate, and pelleted at 500 *g* for 5 min in a swinging bucket centrifuge (Eppendorf 5810r). Media was then aspirated, and cells were washed once with 100 μl cold PBS. Finally, PBS was removed and cells were resuspended in 50 μl of tiny‐sci lysis buffer; see (preprint: Martin *et al*, 2021) for details.

Five microliters of each well was then transferred, while preserving orientation, to a 96‐well PCR plate. To each well, we added 0.5 μl of 10 mM dNTPs and 1 μl of 10 μM indexed reverse transcription (RT) primer. Indexed RT, indexed ligation and indexed PCR were all performed as described in preprint: Martin *et al* (2021). For this experiment, a single 96‐well plate of RT primers and a single plate of 96 ligation primers were used. Prior to indexed PCR, 1,000 nuclei were deposited per well. The resulting libraries were purified and sequenced on using the NextSeq 75 cycle high output kit with the following read lengths: 34 bp – R1; 10 bp I1; 48 bp R2. Sequenced reads were demultiplexed, mapped to GRCh38 using STAR, deduplicated, and output as a cell*gene count matrix as described previously (Srivatsan *et al*, 2020). Sample size was chosen such that single cell transcriptomes could be collected from each drug combination such that cells or molecules either matched or were in excess of previously collected data.

We generated a novel validation dataset of 32 samples containing combinations of 13 compounds selected from the 188 compounds in sci‐Plex 2 (Srivatsan *et al*, 2020). Drug dose combinations were administered as described previously. Briefly, A549 cells were grown in DMEM media (ThermoFIsher Cat number) supplemented with 10% FBS and 1% Penicillin–Streptomycin. These cells were then seeded at XX cells/well in a 96‐well plate. To minimize the concentration of DMSO in each well, stock compounds from SelleckChem (concentration of 10 mM) were first diluted to 1:10 in DMSO, followed by a 1:10 dilution in PBS. 1 μl of each diluted compound was then added to the corresponding position in a 96‐well plate containing 99 μl of media to achieve a final concentration of 1 μM for each compound. After 24 h of growth at 37°C and 5% CO_2_, media was removed, cells were all washed once with cold PBS and then After the data was filtered cells expressing fewer than 20 genes and gene expressed in fewer than 200 cells, the counts were log‐normalized. The dataset can be found with the GEO accession code — GSE206741.

#### Interpretation of combinatorial genetic interactions

In the case of genetic screening, previous work by Norman *et al* (2019) proposed a set of metrics to annotate and classify gene–gene interactions based on responder genes. Based on this, here we used measured or predicted gene expression differences with respect to control cells (δ), for gene perturbations *a* (δa), *b* (δb) and double perturbations *ab* (δab), to calculate interaction types by similarity between these three expression vectors.

More specifically, to calculate association coefficients, we use the linear regression coefficients $c_{1}$ and $c_{2}$ obtained from the model

$$\mathrm{\delta ab}=\mathrm{\delta a}c_{1}+\mathrm{\delta b}c_{2}$$

To describe interaction modes, we used the following metrics.

1. similarity between predicted and observed values: $\text{dcor}(\mathrm{\delta a}c_{1}+\mathrm{\delta b}c_{2},\mathrm{\delta ab}$).
2. linear regression coefficients: $c_{1}$ and $c_{2}$.
3. magnitude: ${\left(c_{1}^{2}+c_{2}^{2}\right)}^{1/2}$.
4. dominance: $\left|\mathrm{lo}g_{10}\left(c_{1}/c_{2}\right)\right|$.
5. similarity of single transcriptomes: $\text{dcor}\left(a,b\right)$
6. similarity of single to double transcriptomes: $\text{dcor}\left(\left[a,b\right],\mathrm{ab}\right)$.
7. equal contributions: $\frac{\min\left(\text{dcor}\left(a,b\right),\text{dcor}\left(b,\mathrm{ab}\right)\right)}{\max\left(\text{dcor}\left(a,b\right),\text{dcor}\left(a,\mathrm{ab}\right)\right)}$.

Following clustering and comparison of these metrics across measured and predicted cells, we decided the following rules of thumb to define and annotate interaction modes:

1. epistatic: $\min\left(\mathrm{abs}\left(c_{1}\right),\mathrm{abs}\left(c_{2}\right)\right)>0.2$ and either (i) ($\mathrm{abs}\left(c_{1}\right)>2\mathrm{abs}\left(c_{2}\right)$) or (ii) ($\mathrm{abs}\left(c_{2}\right)>2\mathrm{abs}\left(c_{1}\right)$)
2. potentiation: magnitude >1 and abs($\text{dcor}\left(a,b\right)$) – 1 > 0.2.
3. strong synergy (similar phenotypes): magnitude >1 and abs($\text{dcor}\left(\left[a,b\right],\mathrm{ab}\right)$) – 1 > 0.2
4. strong synergy (different phenotypes): magnitude >1 and abs($\text{dcor}\left(a,b\right)$) – 1 > 0.5.
5. additive: abs(magnitude) – 1 < 0.1.
6. redundant: abs($\text{dcor}\left(\left[a,b\right],\mathrm{ab}\right)$) – 1 < 0.2 and abs($\text{dcor}\left(a,b\right)$) – 1 < 0.2

More than one genetic interaction can be suggested from these rules. In those cases, we did not assign any plausible interaction. For visualization purposes, we consider perturbed genes with 50 or more interaction modes reported with other co‐perturbed genes (Appendix Fig S11C).

To visualize differentially expressed genes with the similar response across perturbations (Appendix Fig S11D), we trained a random forest classifier using as prediction labels control, a, b and ab cells, and gene expression as features. We retrieved the top 200 genes from this approach. Then, we annotated the direction (positive or negative) and the magnitude of those changes versus control cells, generating a code for clustering and visualization. To label genes with potential interaction effects, we labeled them if the double perturbation predicted magnitude is 1.5× times or higher than the best value observed in single perturbations.

## Author contributions

**Mohammad Lotfollahi:** Conceptualization; data curation; software; formal analysis; visualization; methodology; writing – original draft; project administration; writing – review and editing. **Anna Klimovskaia Susmelj:** Conceptualization; software; formal analysis; methodology; writing – original draft; writing – review and editing. **Carlo De Donno:** Data curation; software; formal analysis; visualization; writing – original draft; writing – review and editing. **Leon Hetzel:** Software; formal analysis; writing – original draft; writing – review and editing. **Yuge Ji:** Data curation; formal analysis; visualization; writing – original draft; writing – review and editing. **Ignacio L Ibarra:** Data curation; formal analysis; visualization; writing – review and editing. **Sanjay R Srivatsan:** Validation; methodology; writing – review and editing. **Mohsen Naghipourfar:** Software; writing – review and editing. **Riza M Daza:** Validation; methodology; writing – review and editing. **Beth Martin:** Validation; writing – review and editing. **Jay Shendure:** Validation; writing – review and editing. **Jose L McFaline‐Figueroa:** Validation; writing – review and editing. **Pierre Boyeau:** Software. **F Alexander Wolf:** Data curation. **Nafissa Yakubova:** Supervision; project administration; writing – review and editing. **Stephan Günnemann:** Writing – review and editing. **Cole Trapnell:** Supervision; validation; methodology; writing – review and editing. **David Lopez‐Paz:** Conceptualization; software; supervision; methodology; writing – original draft; writing – review and editing. **Fabian J Theis:** Conceptualization; supervision; funding acquisition; validation; writing – original draft; project administration; writing – review and editing.

## Disclosure and competing interests statement

ML consults for Santa Ana Bio, Inc. FJT consults for Immunai Inc., Singularity Bio B.V., CytoReason Ltd, Cellarity and Omniscope Ltd, and has ownership interest in Dermagnostix GmbH and Cellarity. FAW has ownership interest in Cellarity, Inc. CD is a full‐time employee of Immunai, Inc. FJT is an editorial advisory board member. This has no bearing on the editorial consideration of this article for publication.

## Supporting information



Appendix

## Data Availability

All datasets analyzed in this manuscript are public and have been published in other papers. We have referenced them in the manuscript and made them available at https://github.com/theislab/cpa‐reproducibility/tree/main/notebooks. In Addition, to that, an open‐source implementation of the code is also available at https://github.com/theislab/cpa.
