# Supplementary material for: Predicting cellular responses to complex perturbations in high‐throughput screens
Source: Mol Syst Biol. 2023 May 8;19(6):e11517. doi: 10.15252/msb.202211517 (PMC10258562; doi:10.15252/msb.202211517)
Supplement: Supplementary file 1 — Appendix [file MSB-19-e11517-s002.pdf]

Supplementary Information

Predicting cellular responses to complex perturbations in high-throughput screens

Mohammad Lotfollahi [1,3,⋆], Anna Klimovskaia Susmelj [2,8,⋆], Carlo De Donno [1,3,⋆], Leon Hetzel [1,13], Yuge Ji [1,3], Ignacio L. Ibarra [1], Sanjay R. Srivatsan [4], Mohsen Naghipourfar [11], Riza M. Daza [4], Beth Martin [4], Jay Shendure [4,5,6,7], Jose L. McFaline-Figueroa [12], Pierre Boyeau [10], F. Alexander Wolf [1,○], Nafissa Yakubova [2], Stephan Günnemann [14], Cole Trapnell [4,5,6], David Lopez-Paz [2], Fabian J. Theis [1,3,4,9‡]

1 Helmholtz Center Munich – German Research Center for Environmental Health, Institute of Computational Biology, Neuherberg, Munich, Germany.
2 Meta AI, 6 Rue Ménars, Paris, 75002, France.
3 School of Life Sciences Weihenstephan, Technical University of Munich, Munich, Germany.
4 Department of Genome Sciences, University of Washington, Seattle, WA, USA.
5 Brotman Baty Institute for Precision Medicine, Seattle, WA, USA.
6 Allen Discovery Center for Cell Lineage Tracing, Seattle, WA, USA.
7 Howard Hughes Medical Institute, Seattle, WA, USA.
8 Swiss Data Science Center, Zurich, Switzerland.
9 Wellcome Trust Sanger Institute, Wellcome Genome Campus, Hinxton, Cambridgeshire, UK.
10 Department of Electrical Engineering and Computer Sciences, University of California, Berkeley, USA.
11 Department of Bioengineering, University of California, Berkeley, USA.
12 Department of Biomedical Engineering, Columbia University, New York, NY, USA.
13 Department of Mathematics, Technical University of Munich, Munich, Germany.
14 Department of Computer Science, Technical University of Munich, Munich, Germany.

⋆ These authors contributed equally to the work.
○ Present address: Lamin Labs, Munich.
‡ Correspondence to fabian.theis@helmholtz-muenchen.de

List of Figures

S1 Appendix Figure S1: Extended architecture details for CPA. 3
S2 Appendix Figure S2: CPA disentangles perturbation and covariate information from the basal latent space. 4
S3 Appendix Figure S3: CPA learns cell type specific perturbation effects after IFN-β stimulation of PBMCs. 5
S4 Appendix Figure S4: CPA learns response to various dosages of drug perturbations. . 6
S5 Appendix Figure S5: CPA models the gene response to various dosages of drug perturbations. 7
S6 Appendix Figure S6: CPA learns trends of gene expression across time points. 8
S7 Appendix Figure S7: Performance comparison between CPA and scGen on sciPlex3 . 8
S8 Appendix Figure S8: Comparison of CPA perturbation space with gene expression embedding on Norman et al. 9
S9 Appendix Figure S9: Performance evaluation for CPA combinatorial predictions. . . 10
S10 Appendix Figure S10: Detailed performance evaluation for combinatorial predictions scenario. 11
S11 Appendix Figure S11: Gene-gene interaction insights revealed from genetic perturbation predictions using CPA. 12
S12 Appendix Figure S12: CPA uncertainty estimation. 13

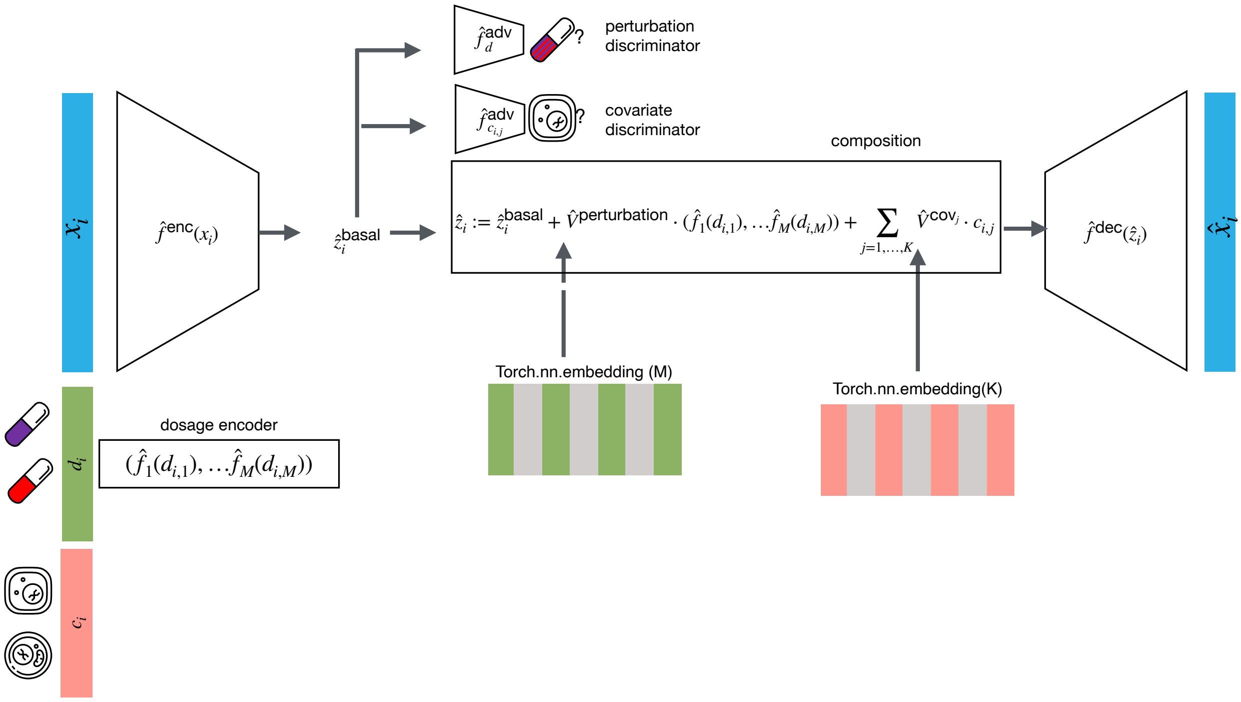

Appendix Figure S1: **Extended architecture details for CPA.**

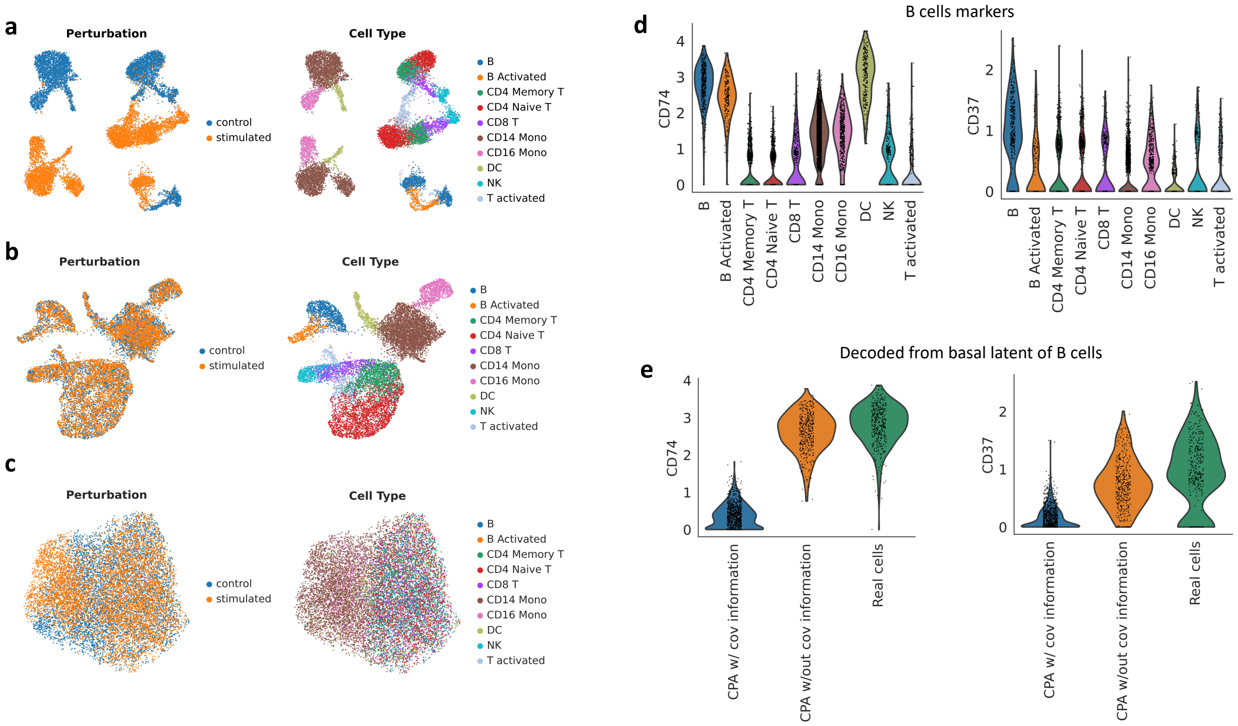

Appendix Figure S2: **CPA disentangles perturbation and covariate information from the basal latent space.** **(a)** UMAP visualization of Kang *et al.* dataset colored by perturbation and cell type. **(b)** Basal latent representation of the data obtained with a CPA model trained using only perturbation information (no cell type label). It is possible to see how the perturbations are mixed but the cell type information is retained. **(c)** Basal latent representation of the dataset obtained with a CPA model trained using both perturbation and cell type information. Both sources of variation are mixed. **(d)** CD74 and CD37 are marker genes of B cells. **(e)** CD74 and CD37 expression of decoded basal B cells. The model trained including cell type labels correctly removes cell-type related information, on the opposite the model trained using only perturbation labels does not disentangle cell type information.

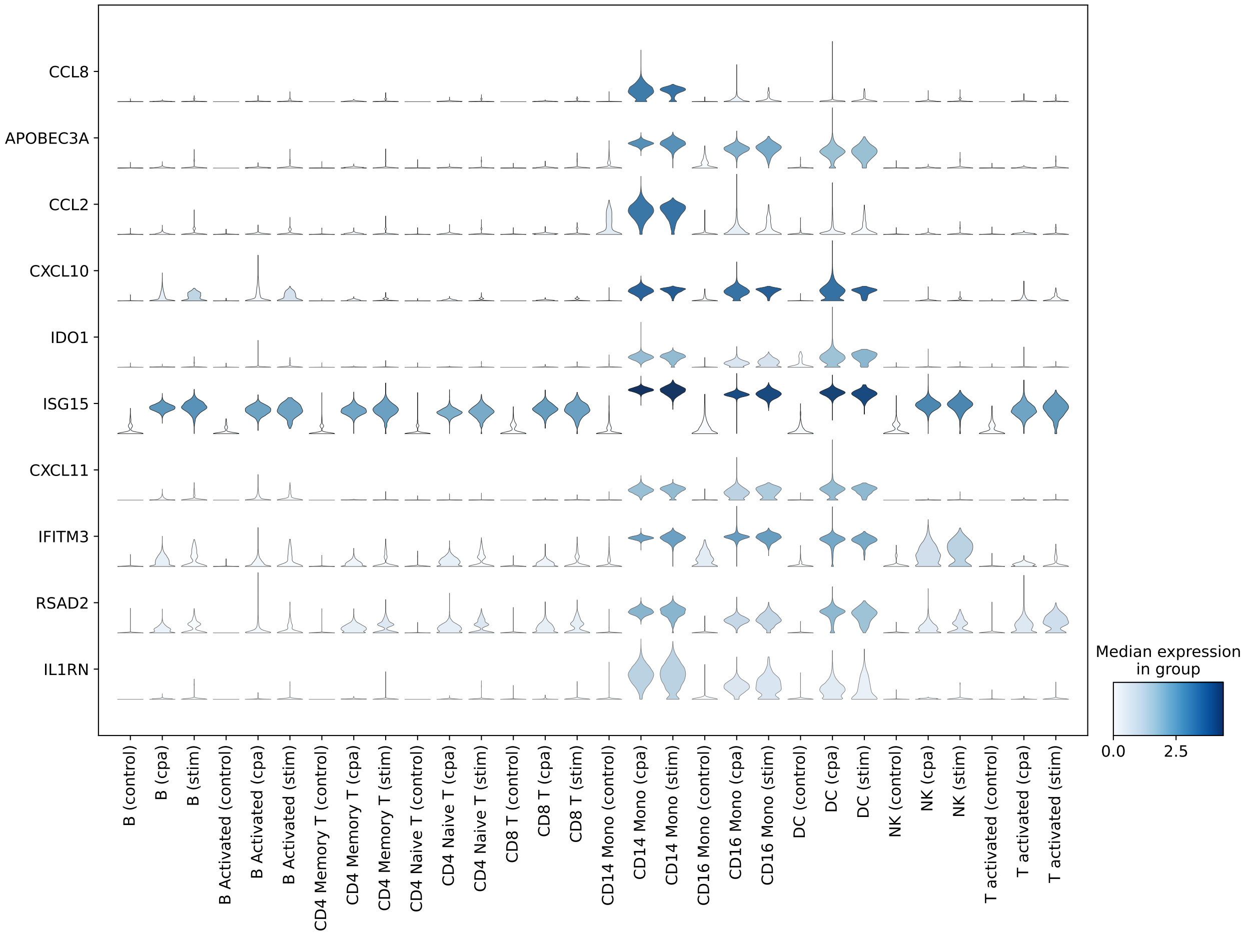

Appendix Figure S3: **CPA learns cell type specific perturbation effects after IFN-β stimulation of PBMCs.** Violin plot for top 10 cell-type specific genes after IFN-β stimulation as previously reported in [1] across, control, real stimulated (stim) and CPA stimulated (cpa) for different cell types. Vertical axis: expression distribution for top specific genes. Horizontal axis: control, real and predicted gene expression via CPA across various cell types.

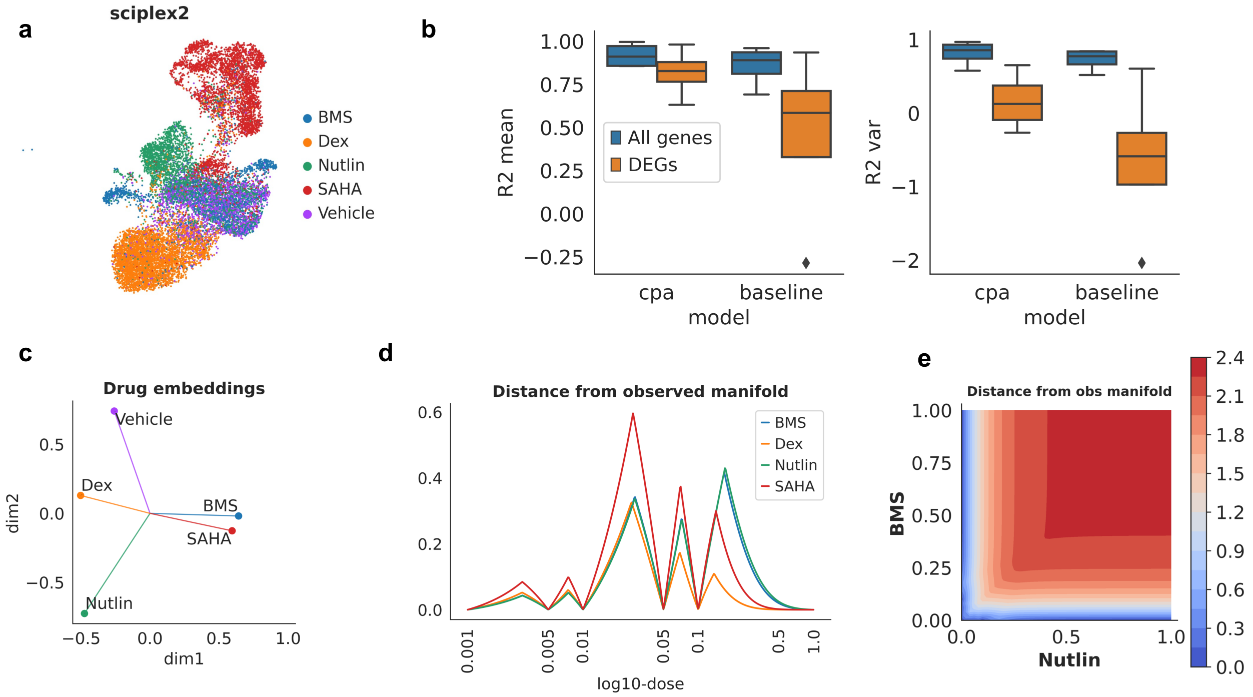

Appendix Figure S4: **CPA learns response to various dosages of drug perturbations. (a)** UMAP representation of the sciplex2 dataset used to fit the model. A549 cells were perturbed with four different drugs at 7 distinct dosages. **(b)** OOD prediction performance comparison between CPA and the random baseline. R2 for mean and variance, and for all genes and only DEGs is reported. **(c)** Kernel PCA (cosine kernel) visualization of the perturbation embeddings obtained after fitting the model to the dataset. **(d)** Distance from the observed manifold for the four different perturbations across dosages. The labeled normalized dosages on the x-axis represent the dosages sampled in the original dataset. The distance increases between these dosage values since these conditions were not observed during training. **(e)** Distance from the observed manifold in the case of combination of two perturbations. Since no combinations are observed during training, these will be farther from the original manifold and are therefore to be considered harder predictions for the model.

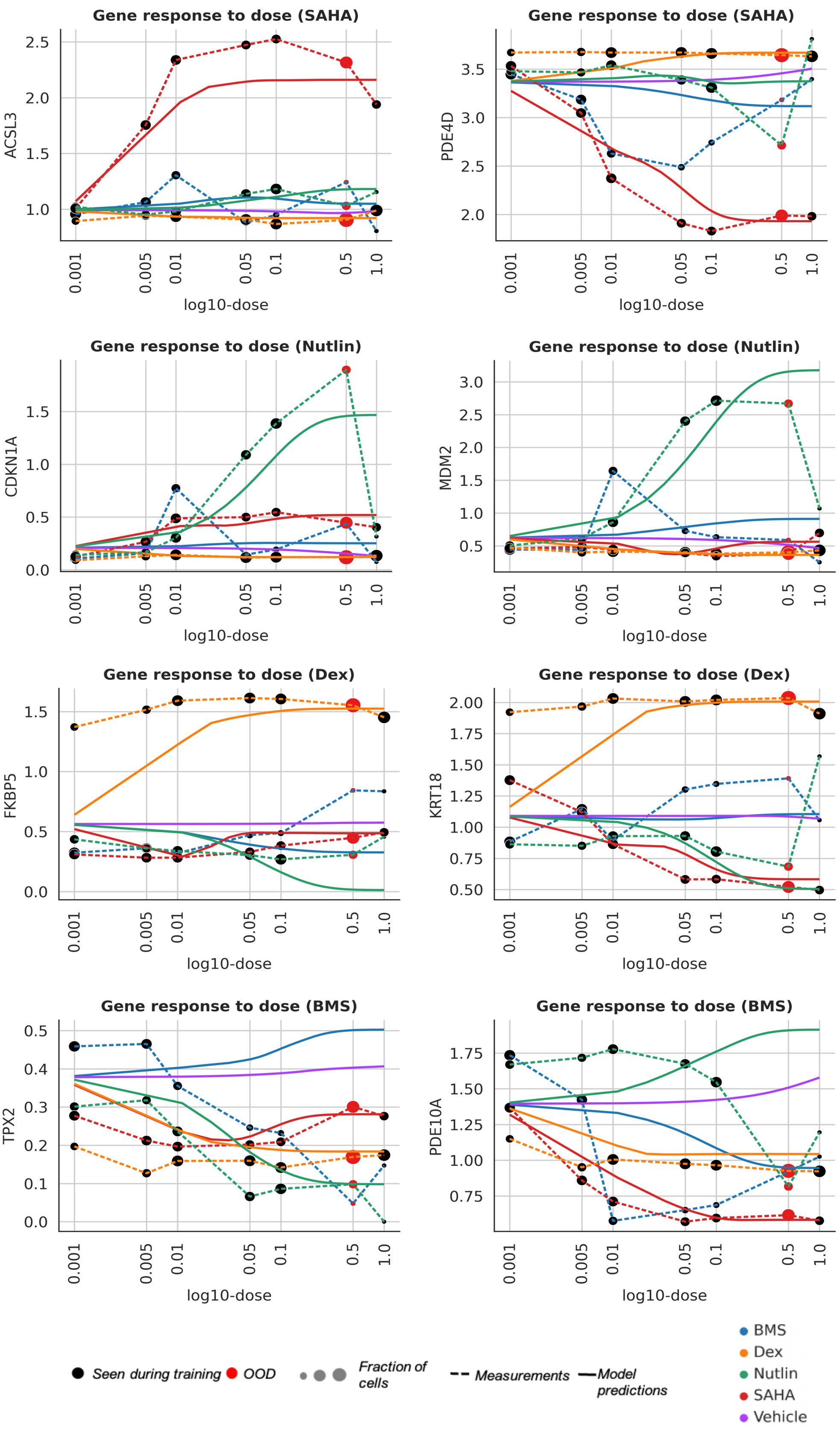

Appendix Figure S5: **CPA models the gene response to various dosages of drug perturbations.** Observed and inferred gene response for the top 2 DEGs for each perturbation across dosages. The dashed lines represent the observed expression pattern for the different perturbation. The full lines show the expression pattern obtained using CPA for inference. The title of the plots indicates for which perturbation the genes were the main responders.

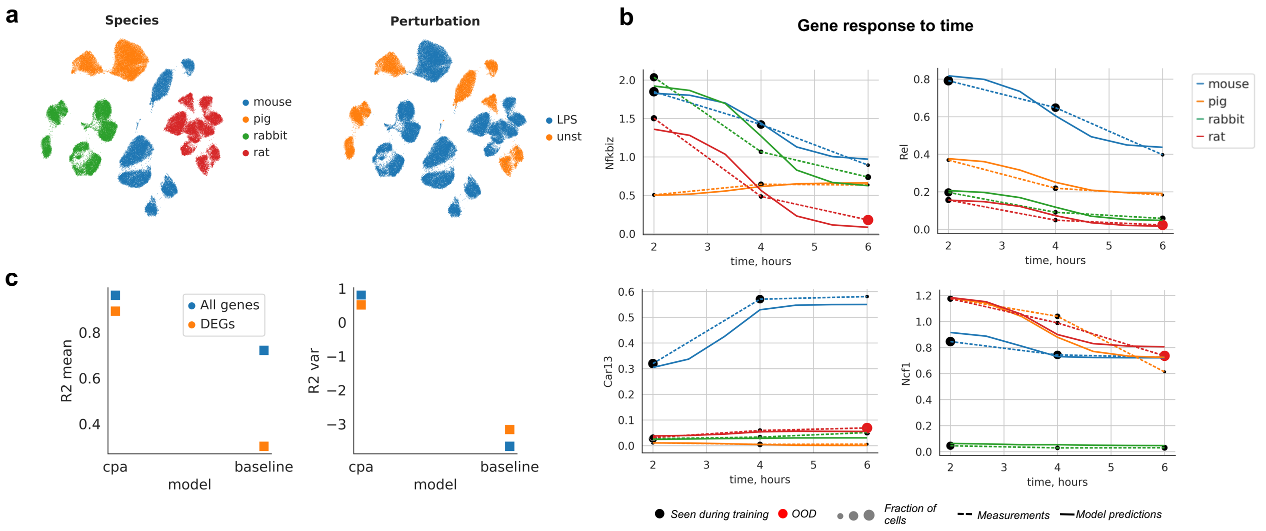

Appendix Figure S6: **CPA learns trends of gene expression across time points. (a)** UMAP visualization of the cross-species dataset used for the experiment [2]. Phagocytes from four different species were treated with LPS and collected at 4 different time points (0 (control), 2, 4, 6 hours). **(b)** Trends of gene expressions across time points for the different species. The full lines are expressions inferred by CPA, dashed lines show the real gene expression. The genes were selected from those indicated in the manuscript of the original study. **(c)** OOD performance of CPA compared to a baseline of a randomly selected subset of the dataset. Rat 6hrs was selected as OOD condition. CPA models successfully both mean and variance of the whole gene vector and DEGs.

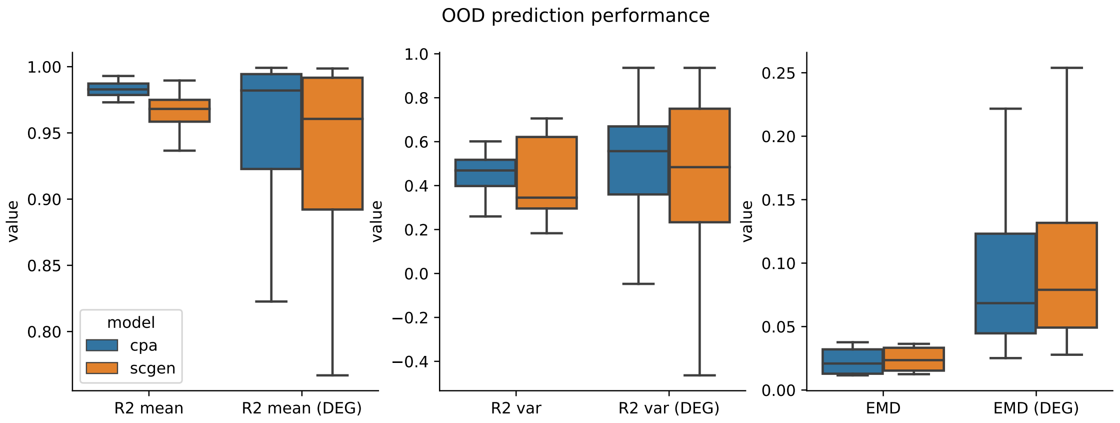

Appendix Figure S7: **Performance comparison between CPA and scGen on sciPlex3**. Comparison or R2 scores (the higher is better) of mean and variance, and EMD (Wasserstein's distance, the lower is better) for OOD conditions between CPA and scGen on the sciPlex3 dataset. To compare with scGen, which can not model dosage, we restricted the dataset to only control and second-highest dosage cells. CPA predictions are more accurate than those obtained using scGen in all the proposed metrics.

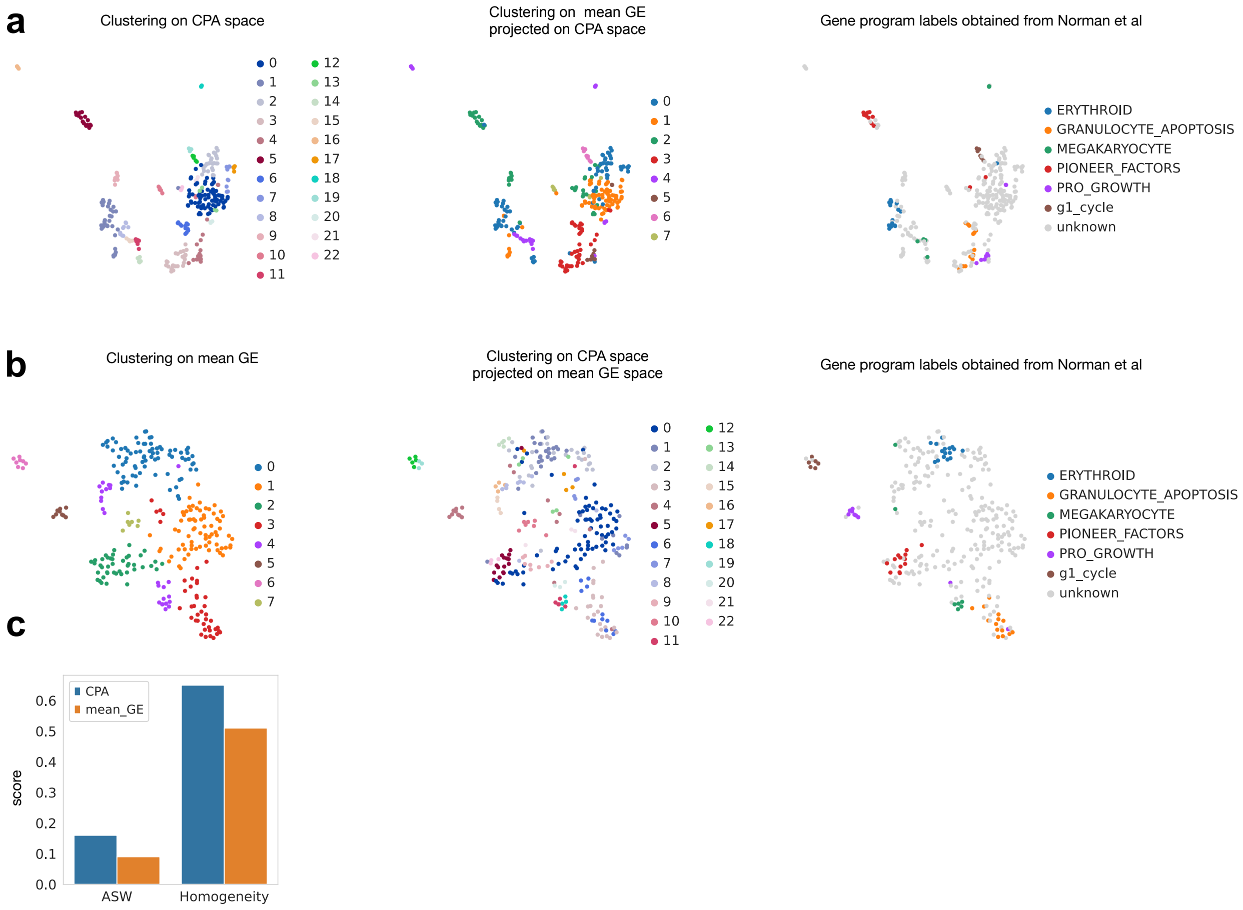

Appendix Figure S8: **Comparison of CPA perturbation space with gene expression embedding on Norman et al. (a-b)** Leiden clustering results on CPA (a) and mean gene expression for each perturbation (b) colored by recovered clusters and gene programs annotations assigned to each perturbation by Norman et al. The unknown cluster was not originally labelled by original authors. **(c)** Comparison of clustering quality according to average silhouette width (ASW) and Homogeneity scores using original perturbation labels from Norman et al.

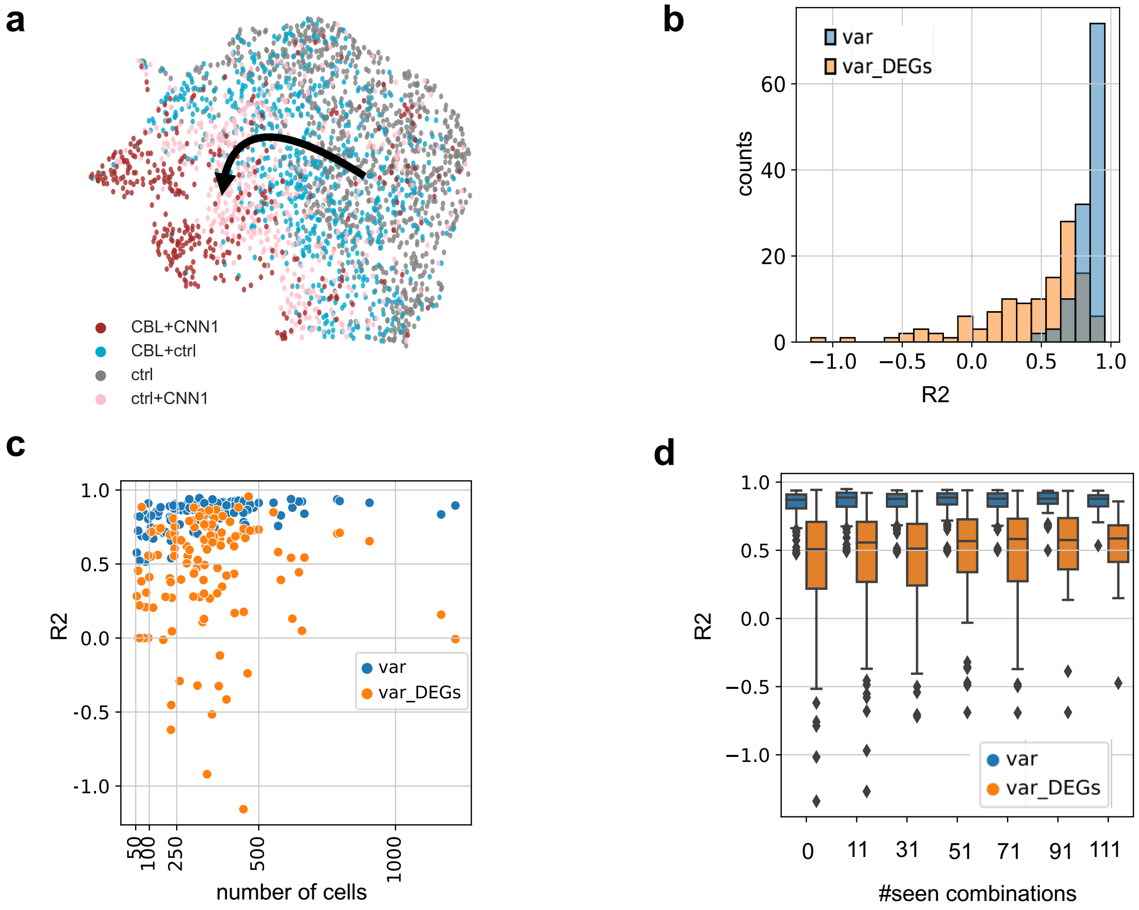

Appendix Figure S9: **Performance evaluation for CPA combinatorial predictions.** **(a)** UMAP representation of control (ctrl), singly perturbed (CBL+ctrl, ctrl+CNN1) and doubly (CBL+CNN1) perturbed cells. **(b)** R^2 scores for all genes (black) or top 100 DEGs (orange) for the prediction of all 131 combinations in the data by training 13 different models and leaving out $\approx$ 10 combinations each time. **(c)** Scatter plots of number of samples in the real data for each combination (x-axis) versus R^2 values for the variance of predicted and real for that combination **(d)** Box-plots of R^2 values for variance for predicted and real cells while increasing the number of combinations seen during training.

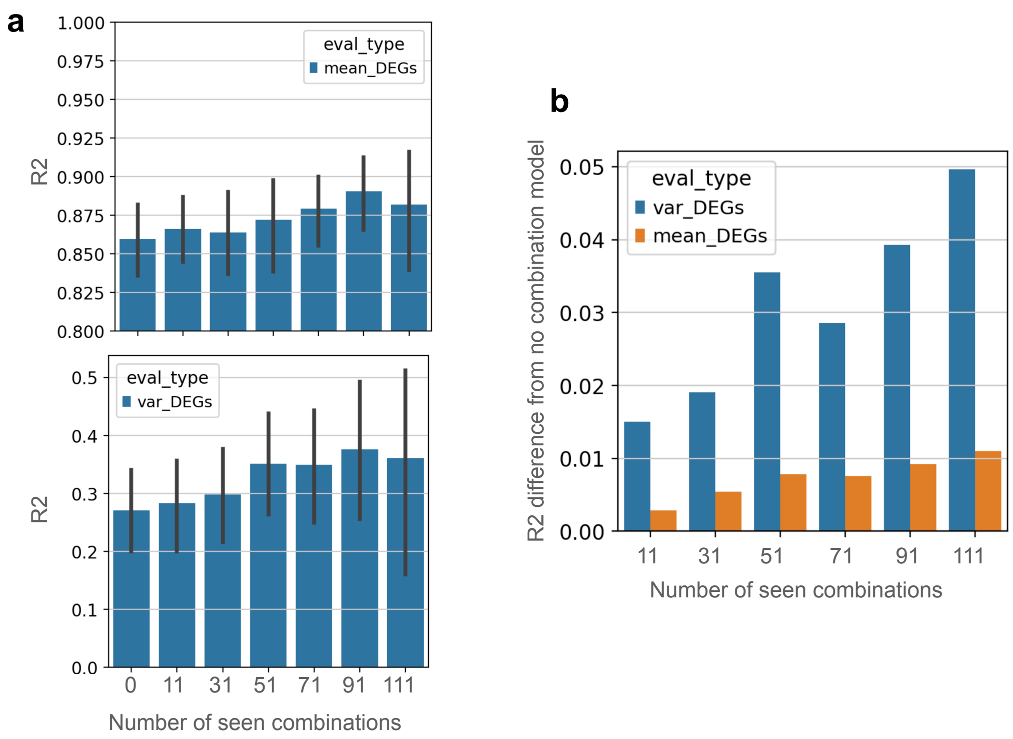

Appendix Figure S10: **Detailed performance evaluation for combinatorial predictions scenario. (a)** Mean and variance estimation prediction for top 100 DEGs across all 131 combinations in the data by training 13 different models and leaving out $\approx$ 10 combinations each time. **(b)** Accuracy difference between models trained with combination data and a model when no combination data is observed. The Y axis shows the difference between mean R2 for models which have seen combinations in training (11, 31, 51, 71, 91,111) and the model with no combination in the training (0 in **a**) for these 20 combinations.

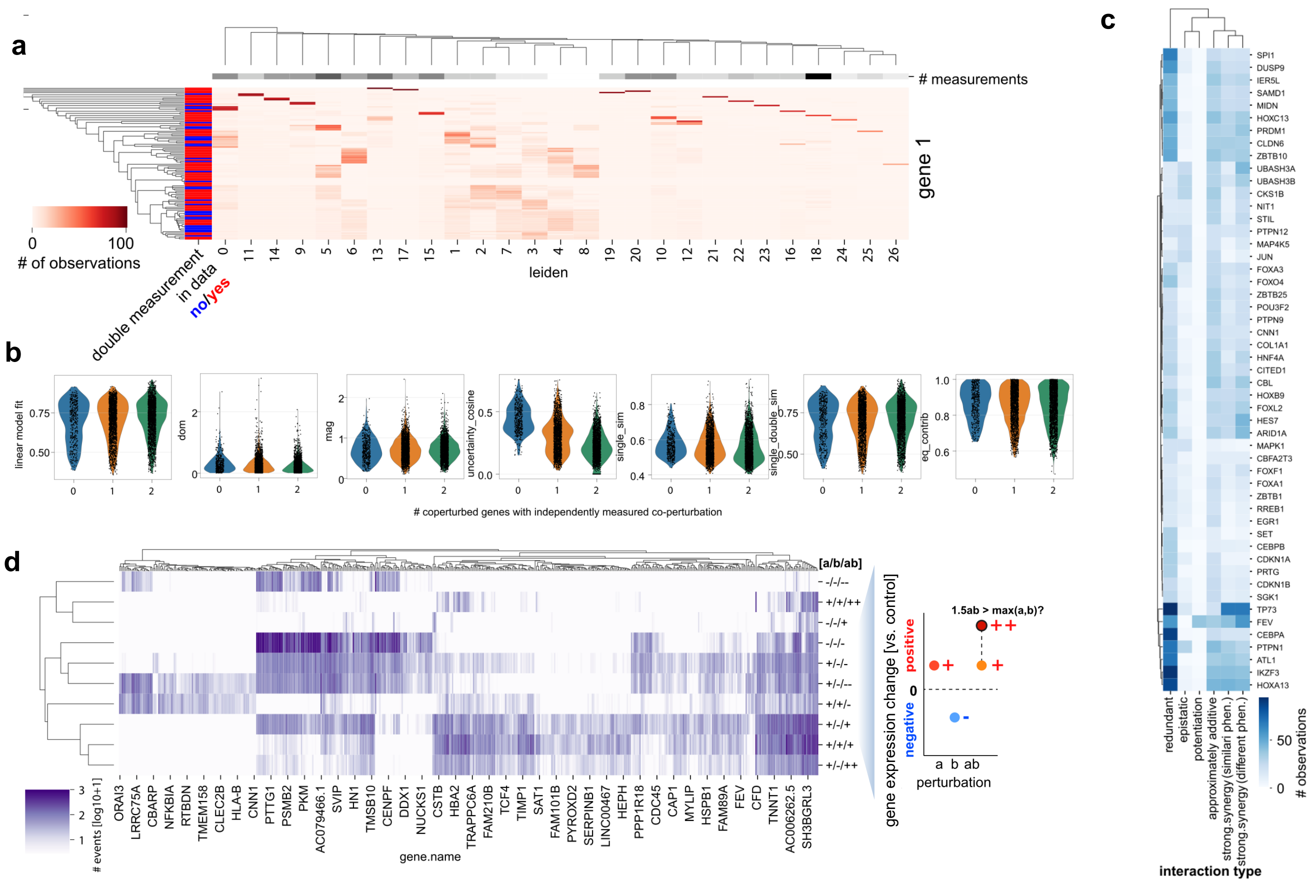

Appendix Figure S11: **Gene-gene interaction insights revealed from genetic perturbation predictions using CPA. (a)** Number of single gene observations in leiden clusters for generated measurements (here 5,160 points out of 5,613 points are used for calculations) (from Figure 4i). Several leiden clusters contain a prevalence for one perturbed gene. The majority of genes without measured double perturbations share a limited number of clusters. **(b)** Quality control and interaction metrics to compare gene expression differences between single and double perturbations. Metrics are shown versus the number of genes with a measured double perturbation (zero, one, or both) (See **Methods** for definitions). **(c)** Interaction mode counts predicted for all genes based on interaction metrics (based on [3]). **(d)** (*right*) 500 genes with highest prevalence in differentially expressed genes across datasets, clustered by prevalent response types from single and double perturbations. (*left*) Gene expression changes for double perturbations (ab) versus single perturbations (a, b), are compared by direction and magnitude. Positive (+) and negative (-) labels indicate increase/decrease versus control cells, and double positive/negative (++/- -) indicate values higher than 1.5 times the highest comparable value in single perturbations.

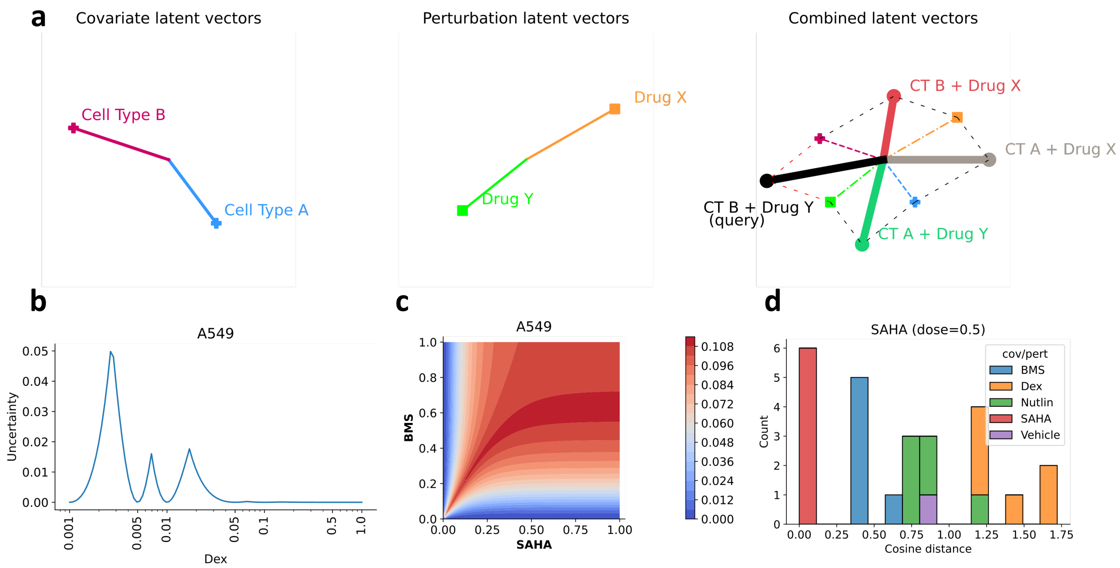

Appendix Figure S12: **CPA uncertainty estimation.** **(a)** Schematic representation of the steps involved in uncertainty estimation in the case of a dataset with two cell types and two drugs (single dosage per drug). The covariate and perturbation latent vectors are summed in order to generate the set of combinations in the training set. The distances from the query vector and all the vectors in the set are then computed. The closest distance is used as a proxy for uncertainty in the prediction of the model. **(b)** Example of uncertainty across dosages of Dexamethasone in the sci-Plex 2 dataset. The ticks on the x-axis (log-scaled) indicate dosages seen at training time for which the uncertainty is 0. The dosages were min-max normalized. **(c)** 2D plot of uncertainty across dosages (min-max normalized) of two different drugs and combinations thereof in the sci-Plex 2 dataset. **(d)** Example histogram of cosine distances between the SAHA (dose=0.5) and the vectors in the set of training perturbations. The distribution shows that training vectors belonging to the same perturbation but with different dosages have the lowest uncertainties, with other drugs being increasingly more distant.

References

[1] Lotfollahi, M., Wolf, F. A. & Theis, F. J. scgen predicts single-cell perturbation responses. Nature methods **16**, 715–721 (2019).

[2] Hagai, T. et al. Gene expression variability across cells and species shapes innate immunity. Nature **563**, 197–202 (2018).

[3] Norman, T. M. et al. Exploring genetic interaction manifolds constructed from rich single-cell phenotypes. Science **365**, 786–793 (2019). Publisher: American Association for the Advancement of Science Section: Research Article.
