# Supplementary material for: Predicting cellular responses to complex perturbations in high‐throughput screens
Source: Mol Syst Biol. 2023 May 8;19(6):e11517. doi: 10.15252/msb.202211517 (PMC10258562; doi:10.15252/msb.202211517)
Supplement: Supplementary file 2 [file MSB-19-e11517-s001.pdf]

molecular systems biology

Predicting cellular responses to complex perturbations in high-throughput screens

Mohammad Lotfollahi, Anna Klimovskaia Susmelj, Carlo De Donno, Leon Hetzel, Yuge Ji, Ignacio Ibarra Del Río, Sanjay Srivatsan, Mohsen Naghipourfar, Riza M. Daza, Beth Martin, Jay Shendure, Jose L. McFaline-Figueroa, Pierre Boyeau, F. Alexander Wolf, Nafissa Yakubova, Stephan Günnemann, Cole Trapnell, David Lopez-Paz, and Fabian Theis
DOI: 10.15252/msb.202211517

Corresponding author(s): Fabian Theis (Fabian.theis@helmholtz-muenchen.de)

Review Timeline:

Submission Date:	21st Dec 22
Editorial Decision:	10th Feb 23
Revision Received:	15th Mar 23
Editorial Decision:	22nd Mar 23
Revision Received:	23rd Mar 23
Accepted:	31st Mar 23

Editor: Maria Polychronidou

Transaction Report:

(Note: With the exception of the correction of typographical or spelling errors that could be a source of ambiguity, letters and reports are not edited. Depending on transfer agreements, referee reports obtained elsewhere may or may not be included in this compilation. Referee reports are anonymous unless the Referee chooses to sign their reports.)

The reviewers' comments and authors' responses are not available with this article, as the initial review process took place with another journal.

Thank you once again for submitting your manuscript "Predicting cellular responses to complex perturbations in high-throughput screens" to Molecular Systems Biology together with your responses to the comments of the reviewers from the other journal. Given that the reviewers appreciated that the presented methodology could be valuable and their reviews were thorough and constructive, we decided to proceed with making a decision based on your responses to these reports rather than reviewing the study from scratch. We contacted reviewers #1 and #2, who reviewed the initial version of the study at the other journal. We specifically raised their attention to the concerns related to the need to:

i) perform experimental validations
ii) include further benchmarking
iii) perform analyses of additional datasets
iv) improve code availability to ensure uptake by the single-cell community
v) improve the accessibility of the findings by editing the text
We clarified that the study would need to be evaluated keeping in mind the scope and editorial requirements of MSB.

We have now heard back from one of the two reviewers who were asked to evaluate your study. Unfortunately, after several reminders we did not manage to obtain a report from reviewer #1. In the interest of time, we have decided to proceed with making a decision based on the comments of reviewer #2. As you will see below, reviewer #2 thinks that all issues have been satisfactorily addressed and is supportive of publication. I am glad to inform you that we have decided to proceed with publishing the study in Molecular Systems Biology, pending a small remaining clarification requested by reviewer #2 and some editorial issues listed below.

Reviewer #2:

The authors have done an admirable job of responding to reviewer comments. The validation with a new drug combination scRNA dataset is quite impressive, and the addition of the chemCPA model further strengthens the paper. My previous comments have been addressed satisfactorily. Overall, despite some rather hostile comments from previous reviewers that seem a bit unfair, this is an outstanding paper that is ready for publication.

The authors did seem to misunderstand one small comment that I raised before. I asked how the CPA predictions on the K562 Perturb-Seq dataset compared to the original authors' predictions. I understand that the goal of the original paper was not to predict gene expression, but rather whether combinatorial perturbations were additive, synergistic, or antagonistic. Since CPA is also used to label the same combinatorial perturbations as synergistic etc. it would be interesting to see how these labels compare with those from the recommender system approach from Norman et al.

Reviewer #2:

The authors have done an admirable job of responding to reviewer comments. The validation with a new drug combination scRNA dataset is quite impressive, and the addition of the chemCPA model further strengthens the paper. My previous comments have been addressed satisfactorily. Overall, despite some rather hostile comments from previous reviewers that seem a bit unfair, this is an outstanding paper that is ready for publication.

The authors did seem to misunderstand one small comment that I raised before. I asked how the CPA predictions on the K562 Perturb-Seq dataset compared to the original authors' predictions. I understand that the goal of the original paper was not to predict gene expression, but rather whether combinatorial perturbations were additive, synergistic, or antagonistic. Since CPA is also used to label the same combinatorial perturbations as synergistic etc. it would be interesting to see how these labels compare with those from the recommender system approach from Norman et al.

We thank the reviewer for constructive feedback and comments that led to major improvements in the paper. Regarding the comment on the interaction labels, we did not use CPA to directly annotate the interaction type but **only** to predict gene expression response. In a subsequent step, to annotate interaction types for unseen predictions, we used the same linear model (not CPA) proposed in the original publication to assign interaction types, based on coefficients obtained from our predictions. This linear model was applied in the original study on mean gene expression vectors obtained from the data we used for training. Therefore we do not have labels to compare against the out-of-distribution data we generated. We added a short sentence in the discussion section to clarify this again.

Regarding the recommender system approach from Norman et al., this is another type of model, which is not applicable here since it takes GI scores from sampled perturbations as input, and infers the remaining GI scores using a low-rank matrix approximation. CPA can not be used to predict GI scores similarly in our presented modeling task. This has already been stated in the results section:

> *To investigate the type of interaction between the newly predicted conditions, we compared the differences between double and single perturbations versus control cells and thus annotated their interaction modes adapted from the original publication (Norman et al. 2019).*

Thank you for sending us your revised manuscript. We are now satisfied with the modifications made and we can soon proceed with accepting the study for publication.

Before we formally accept the manuscript for publication, we would ask you to address some remaining editorial issues listed below.

All editorial and formatting issues were resolved by the authors.

Thank you again for sending us your revised manuscript. We are now satisfied with the modifications made and I am pleased to inform you that your paper has been accepted for publication.

EMBO Press Author Checklist

Corresponding Author Name: Fabian Theis
Journal Submitted to: MSB
Manuscript Number: MSB-2022-11517

USEFUL LINKS FOR COMPLETING THIS FORM
The EMBO Journal - Author Guidelines
EMBO Reports - Author Guidelines
Molecular Systems Biology - Author Guidelines
EMBO Molecular Medicine - Author Guidelines

Reporting Checklist for Life Science Articles (updated January
This checklist is adapted from Materials Design Analysis Reporting (MDAR) Checklist for Authors. MDAR establishes a minimum set of requirements in transparent reporting in the life sciences (see Statement of Task: 10.31222/osf.io/9sm4x). Please follow the journal's guidelines in preparing your
Please note that a copy of this checklist will be published alongside your article.

Abridged guidelines for figures
1. Data
The data shown in figures should satisfy the following conditions:
- the data were obtained and processed according to the field's best practice and are presented to reflect the results of the experiments in an accurate and unbiased manner.
- ideally, figure panels should include only measurements that are directly comparable to each other and obtained with the same assay.
- plots include clearly labeled error bars for independent experiments and sample sizes. Unless justified, error bars should not be shown for technical
- if n<5, the individual data points from each experiment should be plotted. Any statistical test employed should be justified.
- Source Data should be included to report the data underlying figures according to the guidelines set out in the authorship guidelines on Data

2. Captions
Each figure caption should contain the following information, for each panel where they are relevant:
- a specification of the experimental system investigated (eg cell line, species name).
- the assay(s) and method(s) used to carry out the reported observations and measurements.
- an explicit mention of the biological and chemical entity(ies) that are being measured.
- an explicit mention of the biological and chemical entity(ies) that are altered/varied/perturbed in a controlled manner.
- the exact sample size (n) for each experimental group/condition, given as a number, not a range;
- a description of the sample collection allowing the reader to understand whether the samples represent technical or biological replicates (including how many animals, litters, cultures, etc.).
- a statement of how many times the experiment shown was independently replicated in the laboratory.
- definitions of statistical methods and measures:

- common tests, such as t-test (please specify whether paired vs. unpaired), simple χ2 tests, Wilcoxon and Mann-Whitney tests, can be unambiguously identified by name only, but more complex techniques should be described in the methods section;

- are tests one-sided or two-sided?
- are there adjustments for multiple comparisons?
- exact statistical test results, e.g., P values = x but not P values < x;
- definition of 'center values' as median or average;
- definition of error bars as s.d. or s.e.m.

Please complete ALL of the questions below.
Select "Not Applicable" only when the requested information is not relevant for your study.

Materials

**Newly Created Materials**	**Information included in the manuscript?**	**In which section is the information available?** (Reagents and Tools Table, Materials and Methods, Figures, Data Availability Section)
New materials and reagents need to be available; do any restrictions apply?	Not Applicable	

**Antibodies**	**Information included in the manuscript?**	**In which section is the information available?** (Reagents and Tools Table, Materials and Methods, Figures, Data Availability Section)
For **antibodies** provide the following information:<br>- Commercial antibodies: RRID (if possible) or supplier name, catalogue number and or/clone number<br>- Non-commercial: RRID or citation	Not Applicable	

**DNA and RNA sequences**	**Information included in the manuscript?**	**In which section is the information available?** (Reagents and Tools Table, Materials and Methods, Figures, Data Availability Section)
**Short novel DNA or RNA including primers, probes**: provide the sequences.	Not Applicable	

**Cell materials**	**Information included in the manuscript?**	**In which section is the information available?** (Reagents and Tools Table, Materials and Methods, Figures, Data Availability Section)
**Cell lines:** Provide species information, strain. Provide accession number in repository **OR** supplier name, catalog number, clone number, and/**OR** RRID.	Yes	It is available in datasets section.
**Primary cultures:** Provide species, strain, sex of origin, genetic modification status.	Not Applicable	
Report if the cell lines were recently **authenticated** (e.g., by STR profiling) and tested for mycoplasma contamination.	Not Applicable	

**Experimental animals**	**Information included in the manuscript?**	**In which section is the information available?** (Reagents and Tools Table, Materials and Methods, Figures, Data Availability Section)
**Laboratory animals or Model organisms:** Provide species, strain, sex, age, genetic modification status. Provide accession number in repository **OR** supplier name, catalog number, clone number, **OR** RRID.	Not Applicable	
**Animal observed in or captured from the field:** Provide species, sex, and age where possible.	Not Applicable	
Please detail **housing and husbandry conditions**.	Not Applicable	

**Plants and microbes**	**Information included in the manuscript?**	**In which section is the information available?** (Reagents and Tools Table, Materials and Methods, Figures, Data Availability Section)
**Plants:** provide species and strain, ecotype and cultivar where relevant, unique accession number if available, and source (including location for collected wild specimens).	Not Applicable	
**Microbes:** provide species and strain, unique accession number if available, and source.	Not Applicable	

**Human research participants**	**Information included in the manuscript?**	**In which section is the information available?** (Reagents and Tools Table, Materials and Methods, Figures, Data Availability Section)
If collected and within the bounds of privacy constraints report on age, sex and gender or ethnicity for all study participants.	Not Applicable	

**Core facilities**	**Information included in the manuscript?**	**In which section is the information available?** (Reagents and Tools Table, Materials and Methods, Figures, Data Availability Section)

If your work benefited from core facilities, was their service mentioned in the acknowledgments section?	Not Applicable	

Design

**Study protocol**	**Information included in the manuscript?**	**In which section is the information available?** (Reagents and Tools Table, Materials and Methods, Figures, Data Availability Section)
If study protocol has been **pre-registered, provide DOI in the manuscript**. For clinical trials, provide the trial registration number **OR** cite DOI.	Not Applicable	
Report the **clinical trial registration number** (at ClinicalTrials.gov or equivalent), where applicable.	Not Applicable	

**Laboratory protocol**	**Information included in the manuscript?**	**In which section is the information available?** (Reagents and Tools Table, Materials and Methods, Figures, Data Availability Section)
Provide DOI OR other citation details if **external detailed step-by-step protocols** are available.	Yes	It is available in datasets section.

**Experimental study design and statistics**	**Information included in the manuscript?**	**In which section is the information available?** (Reagents and Tools Table, Materials and Methods, Figures, Data Availability Section)
Include a statement about **sample size** estimate even if no statistical methods were used.	Yes	It is available in datasets section.
Were any steps taken to minimize the effects of subjective bias when allocating animals/samples to treatment (e.g. **randomization procedure**)? If yes, have they been described?	Not Applicable	
Include a statement about **blinding** even if no blinding was done.	Not Applicable	
Describe **inclusion/exclusion criteria** if samples or animals were excluded from the analysis. Were the criteria pre-established?<br><br>If sample or data points were omitted from analysis, report if this was due to attrition or intentional exclusion and provide justification.	Yes	It is available in datasets section.
For every figure, are **statistical tests** justified as appropriate? Do the data meet the assumptions of the tests (e.g., normal distribution)? Describe any methods used to assess it. Is there an estimate of variation within each group of data? Is the variance similar between the groups that are being statistically compared?	Not Applicable	

**Sample definition and in-laboratory replication**	**Information included in the manuscript?**	**In which section is the information available?** (Reagents and Tools Table, Materials and Methods, Figures, Data Availability Section)
In the figure legends: state number of times the experiment was **replicated** in laboratory.	Not Applicable	
In the figure legends: define whether data describe **technical or biological replicates**.	Not Applicable	

Ethics

**Ethics**	**Information included in the manuscript?**	**In which section is the information available?** (Reagents and Tools Table, Materials and Methods, Figures, Data Availability Section)
Studies involving **human participants**: State details of **authority granting ethics approval** (IRB or equivalent committee(s), provide reference	Not Applicable	
Studies involving **human participants**: Include a statement confirming that **informed consent** was obtained from all subjects and that the experiments	Not Applicable	
Studies involving **human participants:** For publication of **patient photos**, include a statement confirming that consent to publish was obtained.	Not Applicable	
Studies involving experimental **animals**: State details of **authority granting ethics approval** (IRB or equivalent committee(s), provide reference number for approval. Include a statement of compliance with ethical regulations.	Not Applicable	
Studies involving **specimen and field samples:** State if relevant **permits** obtained, provide details of authority approving study; if none were required, explain why.	Not Applicable	

**Dual Use Research of Concern (DURC)**	**Information included in the manuscript?**	**In which section is the information available?** (Reagents and Tools Table, Materials and Methods, Figures, Data Availability Section)
Could your study fall under dual use research restrictions? Please check biosecurity documents and list of **select agents and toxins** (CDC): https://www.selectagents.gov/sat/list.htm	Not Applicable	
If you used a select agent, is the security level of the lab appropriate and reported in the manuscript?	Not Applicable	
If a study is subject to dual use research of concern regulations, is the name of the **authority granting approval and reference number** for the regulatory approval provided in the manuscript?	Not Applicable	

Reporting

The MDAR framework recommends adoption of discipline-specific guidelines, established and endorsed through community initiatives. Journals have their own policy about requiring specific guidelines and recommendations to complement MDAR.

**Adherence to community standards**	**Information included in the manuscript?**	**In which section is the information available?** (Reagents and Tools Table, Materials and Methods, Figures, Data Availability Section)
State if relevant guidelines or checklists (e.g., **ICMJE, MIBBI, ARRIVE, PRISMA**) have been followed or provided.	Not Applicable	
For **tumor marker prognostic studies,** we recommend that you follow the **REMARK** reporting guidelines (see link list at top right). See author	Not Applicable	
For **phase II and III randomized controlled trials**, please refer to the **CONSORT** flow diagram (see link list at top right) and submit the CONSORT checklist (see link list at top right) with your submission. See author guidelines, under 'Reporting Guidelines'. Please confirm you have submitted this list.	Not Applicable	

Data Availability

**Data availability**	**Information included in the manuscript?**	**In which section is the information available?** (Reagents and Tools Table, Materials and Methods, Figures, Data Availability Section)
Have **primary datasets** been deposited according to the journal's guidelines (see 'Data Deposition' section) and the respective accession numbers provided in the Data Availability Section?	Yes	It is available in datasets section.
Were **human clinical and genomic datasets** deposited in a public access-controlled repository in accordance to ethical obligations to the patients and to the applicable consent agreement?	Not Applicable	It is available in datasets section.
Are **computational models** that are central and integral to a study available without restrictions in a machine-readable form? Were the relevant accession numbers or links provided?	Yes	It is available in datasets section.
If publicly available data were reused, provide the respective **data citations in the reference list**.	Yes	It is available in datasets section.
